# Accuracy of upper respiratory tract samples to diagnose *Mycobacterium tuberculosis*: a systematic review and meta-analysis

**DOI:** 10.1016/S2666-5247(23)00190-8

**Published:** 2023-10

**Authors:** Helen R Savage, Hannah M Rickman, Rachael M Burke, Maria Lisa Odland, Martina Savio, Beate Ringwald, Luis E Cuevas, Peter MacPherson

**Affiliations:** a Department of Clinical Sciences, Liverpool School of Tropical Medicine, Liverpool, UK; b The LIGHT Consortium, Liverpool School of Tropical Medicine, Liverpool, UK; c Clinical Research Department, London School of Hygiene & Tropical Medicine, London, UK; d Public Health Group, Malawi-Liverpool-Wellcome Programme, Blantyre, Malawi; e Department of Public Health and Nursing, Norwegian University of Science and Technology, Trondheim, Norway; f School of Health and Wellbeing, University of Glasgow, Glasgow, UK

## Abstract

**Background:**

Pulmonary tuberculosis due to *Mycobacterium tuberculosis* can be challenging to diagnose when sputum samples cannot be obtained, which is especially problematic in children and older people. We systematically appraised the performance characteristics and diagnostic accuracy of upper respiratory tract sampling for diagnosing active pulmonary tuberculosis.

**Methods:**

In this systematic review and meta-analysis, we searched MEDLINE, Cinahl, Web of Science, Global Health, and Global Health Archive databases for studies published between database inception and Dec 6, 2022 that reported on the accuracy of upper respiratory tract sampling for tuberculosis diagnosis compared with microbiological testing of sputum or gastric aspirate reference standard. We included studies that evaluated the accuracy of upper respiratory tract sampling (laryngeal swabs, nasopharyngeal aspirate, oral swabs, saliva, mouth wash, nasal swabs, plaque samples, and nasopharyngeal swabs) to be tested for microbiological diagnosis of tuberculous (by culture and nucleic acid amplification tests) compared with a reference standard using either sputum or gastric lavage for a microbiological test. We included cohort, case-control, cross-sectional, and randomised controlled studies that recruited participants from any community or clinical setting. We excluded post-mortem studies. We used a random-effects meta-analysis with a bivariate hierarchical model to estimate pooled sensitivity, specificity, and diagnostics odds ratio (DOR; odds of a positive test with disease relative to without), stratified by sampling method. We assessed bias using QUADAS-2 criteria. This study is registered with PROSPERO (CRD42021262392).

**Findings:**

We screened 10 159 titles for inclusion, reviewed 274 full texts, and included 71, comprising 119 test comparisons published between May 13, 1933, and Dec 19, 2022, in the systematic review (53 in the meta-analysis). For laryngeal swabs, pooled sensitivity was 57·8% (95% CI 50·5–65·0), specificity was 93·8% (88·4–96·8), and DOR was 20·7 (11·1–38·8). Nasopharyngeal aspirate sensitivity was 65·2% (52·0–76·4), specificity was 97·9% (96·0–99·0), and DOR was 91·0 (37·8–218·8). Oral swabs sensitivity was 56·7% (44·3–68·2), specificity was 91·3% (CI 81·0–96·3), and DOR was 13·8 (5·6–34·0). Substantial heterogeneity in diagnostic accuracy was found, probably due to differences in reference and index standards.

**Interpretation:**

Upper respiratory tract sampling holds promise to expand access to tuberculosis diagnosis. Exploring historical methods using modern microbiological techniques might further increase options for alternative sample types. Prospective studies are needed to optimise accuracy and utility of sampling methods in clinical practice.

**Funding:**

UK Medical Research Council, Wellcome, and UK Foreign, Commonwealth and Development Office.

## Introduction

Pulmonary disease due to *Mycobacterium tuberculosis* can be challenging to diagnose because of lack of access to testing services, difficulty obtaining samples, and suboptimal sensitivity of tests. In 2021, more than 4·2 million of the estimated 10·6 million people with incident tuberculosis went undiagnosed^1^ and, of those diagnosed with pulmonary tuberculosis, only 63% were bacteriologically confirmed. Bacterial confirmation is important to ensure correct diagnosis and to identify drug resistance so that the most effective treatment regime can be prescribed.^1^

The most common sample used for diagnosis is sputum, which can be tested by smear microscopy, culture, or nucleic acid amplification tests (NAAT) such as Xpert (Cepheid, Sunnyvale, CA, USA) or Truenat (Molbio, Verna, India). In adults, the only alternative to sputum or induced sputum recommended in the WHO consolidated guidelines on tuberculosis^2^ is urine, which is tested using a point-of-care test to detect lipoarabinomannan (ALERE-LAM, Abbott, Chicago, IL, USA) for people with advanced HIV.

Children commonly do not expectorate sputum; therefore, WHO recommends alternative sample types for diagnosis, including induced sputum, gastric aspirate, gastric lavage, nasopharyngeal aspirate, and stool.^3^ Gastric aspirate has a sensitivity of 73% (Xpert MTB/RIF; Cepheid) compared with microbiological reference standard (32% compared with composite reference standard [positive culture or clinical decision to initiate tuberculosis treatment^2^]) but is invasive, requires fasting and early morning testing, and has low caregiver acceptability.^2^, ^3^ Nasopharyngeal aspirate is less invasive; however, it still requires specialist equipment for suction and has moderate caregiver acceptability (as the procedure can be unpleasant for children). The sensitivity of nasopharyngeal aspirate against a microbiological reference standard is 46% (Xpert MTB/RIF).^2^, ^3^ Stool is a newly recommended specimen type in the 2021 WHO guidelines, has high acceptability, and is non-invasive. The sensitivity of stool Xpert MTB/RIF is 61% against microbiological reference standards (16% against clinical reference standards).^2^, ^3^

**Research in context**

**Evidence before this study**

Globally, in 2021, an estimated 4·2 million of 10·6 million people with incident tuberculosis disease went undiagnosed, emphasising the urgent need for new diagnostic methodologies. Most tuberculosis diagnostics are performed on sputum samples, but many people who need tuberculosis tests (such as children, older people, and people with severe illness admitted to hospital) are often unable to produce sputum. Upper respiratory tract sampling for tuberculosis diagnosis was widely investigated in Europe in the early 20th century and in recent years there has been a resurgence of interest in oral sampling, which holds promise to expand non-sputum-based diagnosis.

**Added value of this study**

This study systematically reviewed diagnostic accuracy evaluations of upper respiratory tract sampling for tuberculosis by collating and synthesising literature from the early 20th century to the present day. In the meta-analysis, we found that upper respiratory tract samples have acceptable sensitivity and specificity compared with sputum culture and have the potential to widen access to tuberculosis diagnosis and increase diagnostic yield. However, there was substantial heterogeneity within the study designs and testing methodologies used. This systematic review and meta-analysis provides new evidence to inform the design of much-needed prospective studies on upper respiratory tract samples. For example, studies using newer molecular and culture-based methods to optimise tuberculosis diagnostics that could be capable of meeting WHO Target Product Profiles.

**Implications of all the available evidence**

Upper respiratory tract sampling methodologies for tuberculosis (eg, oral sampling and sampling from the larynx and nasopharynx) might hold promise to expand access to tuberculosis diagnosis, including for people who cannot produce sputum. These sampling strategies can be optimised using modern microbiological techniques to increase access to diagnostics for tuberculosis. Prospective studies are needed to optimise the accuracy and utility of sampling methods in clinical practice.

Alternative sample types that can be processed with both existing and novel tests, are patient centred, and can be collected at the time of consultation, are urgently required. WHO Target Product Profiles have defined the diagnostic accuracy standards for tests to diagnose tuberculosis, and have defined desirable characteristics, including using non-sputum samples such as urine, stool, oral mucosal transudates, saliva, exhaled air, or blood from a fingerstick.^4^

Sampling of the upper respiratory tract (from the mouth and nose, down to the level of the larynx and vocal cords) is minimally invasive and can be performed quickly as an outpatient test, offering the potential to expand access to microbiological tuberculosis diagnosis in those that cannot produce sputum.

We, therefore, set out to systematically appraise the evidence for the performance characteristics and diagnostic accuracy of upper respiratory tract sampling for diagnosing active pulmonary tuberculosis disease. We hypothesised that upper respiratory tract tests would have sufficient accuracy and ease of use to increase access to tuberculosis diagnosis.

## Methods

### Search strategy and selection criteria

The published protocol for this systematic review and meta-analysis is available online. We developed a search strategy with information specialists at the Liverpool School of Tropical Medicine library (Liverpool, UK; appendix p 2). We searched MEDLINE, Cinahl, Web of Science, Global Health, and Global Health Archive from database inception up to Jan 31, 2021, extended to May 27, 2022, and subsequently this search was extended to Dec 6, 2022. Search terms included terms related to tuberculosis, such as *Mycobacterium tuberculosis*, consumption, wasting, pthisis, and Koch's disease as well as terms related to upper respiratory tract sampling, such as oral swab, laryngeal, tonsils, saliva, Waldemeyers ring, pharynx, and pharyngeal.

We included studies that evaluated the accuracy of upper respiratory tract sampling (index tests including laryngeal swabs, nasopharyngeal aspirate, oral swabs, saliva, mouth wash, nasal swabs, plaque samples, and nasopharyngeal swabs) for a microbiological (culture and NAATS, including automated platforms and laboratory PCR) diagnosis of tuberculosis disease compared with a reference standard using either sputum or gastric lavage for a microbiological test. We included cohort, case-control, cross-sectional, and randomised controlled studies (peer-reviewed manuscripts and preprints) that recruited participants from any community or clinical setting. We placed no restrictions on publication language. We excluded studies where the index or reference standard used histological or biomarker-based testing; post-mortem studies; studies in non-human animal species; case reports; clinical guidelines; and studies testing for latent tuberculosis infection (where active tuberculosis disease was not tested for).

Titles and abstracts were imported into a Rayyan.ai database^5^—removing duplicates—and screened by one reviewer (HRS) to exclude titles not related to tuberculosis. A broad search was used to capture as many studies as possible; however, the majority of results were not related to tuberculosis. An R script was used to generate a random subset of 10% of titles, which were checked for agreement by a masked second reviewer (HMR or RMB). The full texts of manuscripts related to tuberculosis were then independently assessed by two reviewers (two of HRS, HMR, and RMB) for inclusion. In cases of disagreement, the third reviewer acted as an arbitrator. Studies that had not been identified as duplicates due to different recorded titles in databases (mainly historical) were removed at full text review.

Manuscripts published in languages other than English were translated prior to full text review and data extraction. Articles in French, Spanish, Italian, Portuguese, German, Norwegian, and Swedish were translated by study authors who are native speakers. Studies in Hungarian and Czech were translated using translation software (Google Translate and DeepL Pro).

Data were extracted using a piloted extraction form. For each included manuscript, HRS extracted data on year, author, country, type of publication, original language, study design, setting, population, swab or device used, technique of sampling, site of sampling, number of samples per participant, timing of swabs, swab preparation, transport, storage, method of testing, number included in analysis, sex, HIV status, median age, and results of index and reference standard tests.

If a manuscript contained multiple index tests, reference tests, or cohorts of patients, each dataset was included as a separate report. As participants might have been included in more than one index test comparison, we reported the number of sample sets included in the analysis for each test comparison undertaken.

### Data synthesis and analysis

We summarised study characteristics and, separately for each upper respiratory tract sampling methodology, graphed forest plots, and calculated the pooled sensitivity and specificity of the index test compared to reference standards using a bivariate hierarchical random effects model, fitted using the lme4 package in R (version 4.2.1) based on the *Cochrane Handbook for systematic reviews of diagnostic test accuracy*.^6^

As heterogeneity was expected, we selected a hierarchical random effects model for meta-analysis.^7^ If sufficient reports were identified, a model was fitted for each index test to give a pooled sensitivity and specificity. We plotted summary receiver operating characteristic (SROC) curves giving a visual indication of variability and heterogeneity. The 95% CI around the summary point indicated the area in which we would expect the sensitivity and specificity of a future study to fall. We performed meta-regression using the two reference standards—sputum and gastric aspirate or lavage—as covariates to compare sensitivity and specificity.

For each index sample type, the diagnostic odds ratio (DOR) was estimated. DOR describes how many times higher the odds of obtaining a positive test result in someone with the target condition are than in someone without the target condition.^8^ We also calculated positive (PLR; how many times more likely positive index test results are with the target condition than without) and negative likelihood ratios (NLR; how many times less likely negative index test results were with the target condition than without).^9^ If studies included children (aged younger than 15 years), a second model using a composite reference standard, including microbiological and clinical cases of pulmonary tuberculosis, was performed (as in the recent WHO consolidated guidelines; see appendix pp 2–4 for full description).^2^, ^3^ This method was not used for studies in adults as data were not available. Data and code to reproduce analysis are available at https://osf.io/9nuvq/. Reports were included in the systematic review if they met eligibility criteria. However, reports were only included in meta-analysis if data were available to compare index tests accuracy to the reference standard (appendix p 5). Risk of bias was assessed using the QUADAS-2 tool.^10^ This study is registered in PROSPERO (CRD42021262392).

### Role of the funding source

The funders of the study had no role in study design, data collection, data analysis, data interpretation, or writing of the report.

## Results

**Figure 1:**
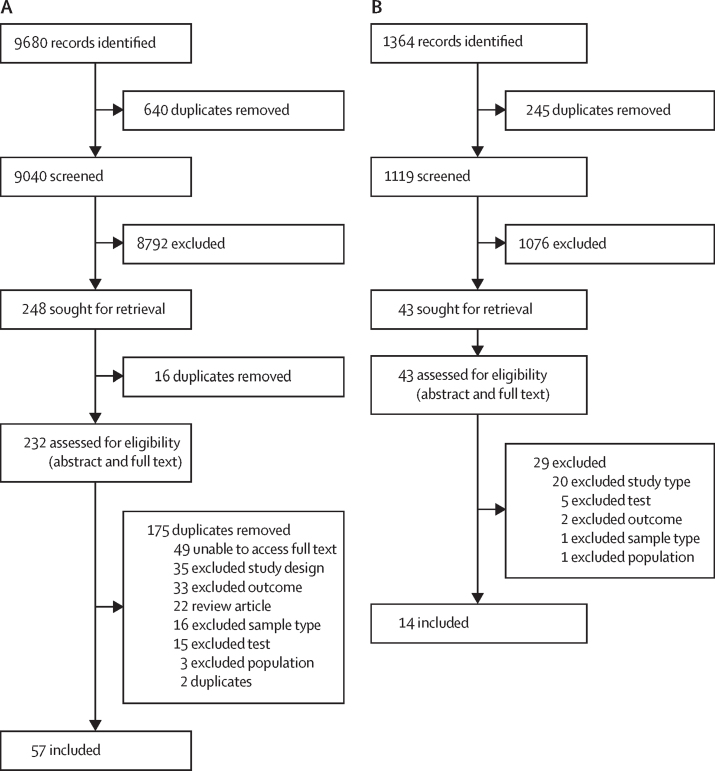
Study selection 71 studies were included overall. (A) First study search date was between Jan 1, 1847, and Jan 1, 2021. (B) Second study search date was between Jan 1, 2021, and May 27, 2022.

9680 studies were identified during the initial search period from database inception to Jan 31, 2021, and a further 1364 were identified when the search was extended to Dec 6, 2022. When duplicates were removed, 10 159 titles (9040 from the initial search and 1119 from the extended search) remained for screening (figure 1). Overall, 71 studies were included in the systematic review, comprising 119 reports on index test comparisons, and 53 were included in meta-analyses. Full details of excluded studies and reasons for exclusion are in the appendix (pp 5–26).

From included manuscripts (appendix pp 26–33 for study characteristics; appendix p 5 includes full methodological details and results by study), we classified types of upper respiratory tract sampling into four groups: laryngeal swabs (32 studies^11^, ^12^, ^13^, ^14^, ^15^, ^16^, ^17^, ^18^, ^19^, ^20^, ^21^, ^22^, ^23^, ^24^, ^25^, ^26^, ^27^, ^28^, ^29^, ^30^, ^31^, ^32^, ^33^, ^34^, ^35^, ^36^, ^37^, ^38^, ^39^, ^40^, ^41^, ^42^), nasopharyngeal aspirate (ten studies^43^, ^44^, ^45^, ^46^, ^47^, ^48^, ^49^, ^50^, ^51^, ^52^), oral swabs (18 studies^53^, ^54^, ^55^, ^56^, ^57^, ^58^, ^59^, ^60^, ^61^, ^62^, ^63^, ^64^, ^65^, ^66^, ^67^, ^68^, ^69^, ^70^), and other (mouthwash three studies,^71^, ^72^, ^73^ nasal swabs one study,^74^ saliva four studies,^75^, ^76^, ^77^, ^78^ and other mucosa or dental samples three studies^79^, ^80^, ^81^). Studies were published between May 13, 1933, and Dec 19, 2022, from South Africa (11 studies), Norway (seven studies), UK (seven studies), Peru (four studies), Uganda (seven studies), Canada (three studies), India (three studies), USA (three studies), Australia (two studies), Germany (two studies), Kenya (two studies), and one study from each of Brazil, Chile, China, Slovakia and Czech Republic, Denmark, Finland, France, Hungary, Italy, Japan, Malawi, Moldova, Mozambique, South Korea, southeast Asia (individual countries not specified), Spain, Sweden, Taiwan, Turkey, and Yemen.

Overall, we included data for 24 899 index test samples from participants in hospitals (29 studies); tuberculosis sanatoria (nine studies); chest clinics (nine studies); tuberculosis hospitals (eight studies); hospital outpatients (seven studies); primary care clinics (three studies); mass screening, asymptomatic, or contact interventions (two studies); outpatient treatment centres (one study); prisons (one study); and unknown settings (five studies). A total of 11 studies were in children aged 16 years or younger, and 60 studies were in adults. In studies that reported demographic data, 1832 (57·7%) of 3173 of participants were male (data from 24 studies), 1341 (42·3%) of 3173 were female, and 722 (19·5%) of 3709 were HIV positive (with 22 of 39 studies conducted after 1981 presenting data on HIV prevalence).

**Figure 2:**
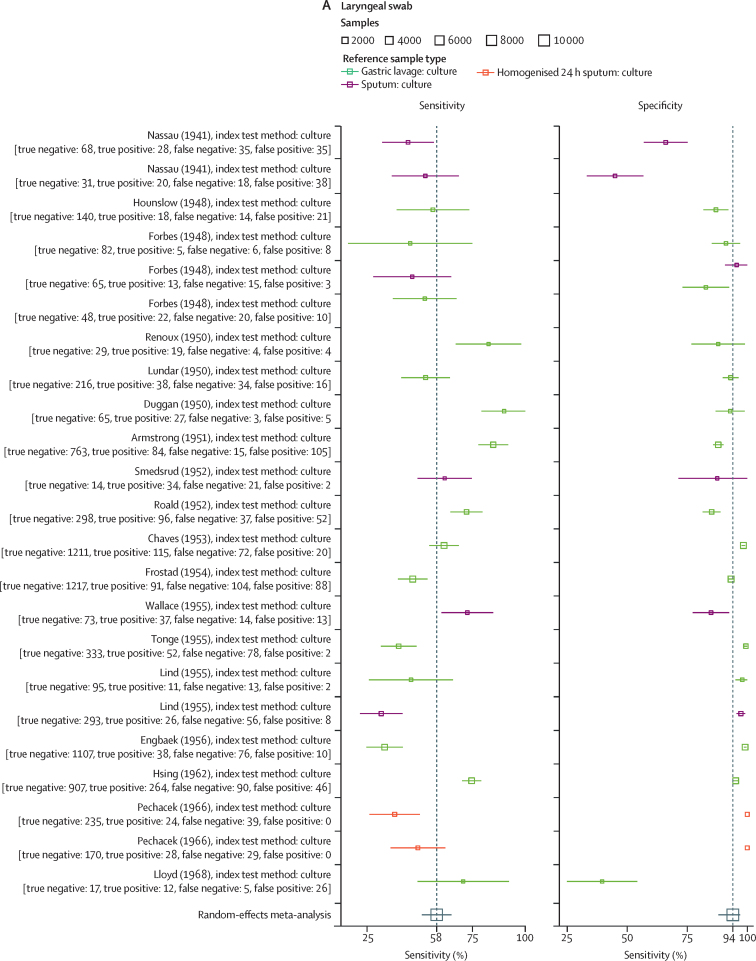

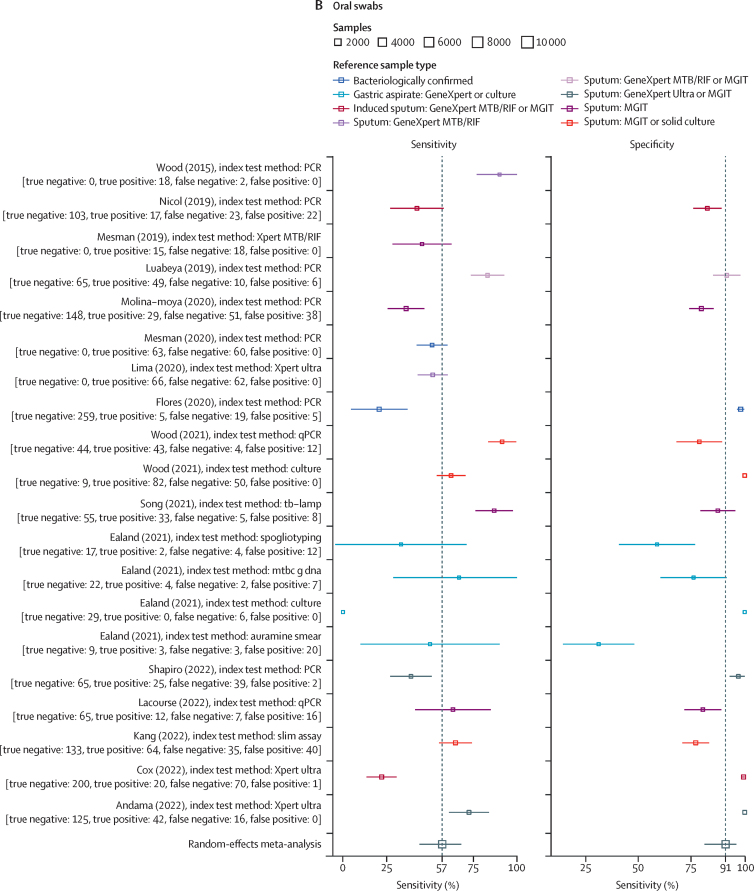

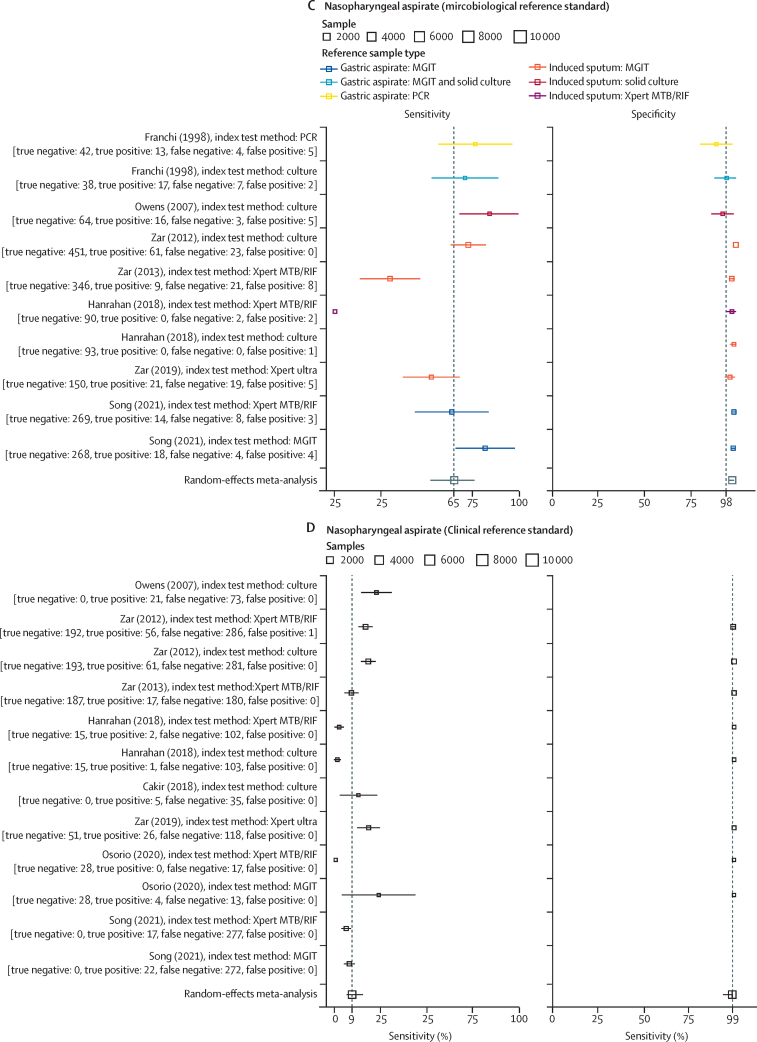
Sensitivity and specificity for alternative sampling methods with random effects meta-analysis (A) Sensitivity and specificity of laryngeal sampling for active pulmonary tuberculosis, with random effects meta-analysis. (B) Sensitivity and specificity of oral swab for active pulmonary tuberculosis, with random effects meta-analysis. (C) Sensitivity and specificity of nasopharyngeal aspirate for active pulmonary tuberculosis, with random effects meta-analysis. (D) Sensitivity and specificity of nasopharyngeal aspirate for active pulmonary tuberculosis when clinical diagnosis used as a reference standard, with random effects meta-analysis. MGIT=Mycobacteria growth indicator tube. TB-LAMP=tuberculosis-loop-mediated isothermal amplification.

41 reports evaluated the accuracy of laryngeal swabs, of which 23 contributed data to the meta-analysis (appendix pp 33–36). Studies were published between May 1, 1941, and March 1, 1968, with sample sizes from ten to 2809 (median 268). Sample preparation methods are detailed in the appendix (pp 5, 26–33). Samples (both index and reference) were cultured using methodologies available at the time of the study. In all studies, the reference test used was culture of either expectorated sputum (eight studies) or gastric lavage (15 studies). Random-effects model estimated sensitivity was 57·8% (95% CI 50·5–65·0), specificity was 93·8% (88·4–96·8), DOR was 20·7 (11·1–38·8), PLR was 9·3 (5·1–17·0), and NLR was 0·45 (0·38–0·53; figure 2A, appendix p 48). A meta-regression to model variability in sample type used as a reference standard showed a statistically significant difference in the modelled specificity—but not sensitivity—when reference sample type was included as a covariate (appendix pp 36–37). The SROC curve (appendix p 48) showed several studies with a considerable distance from the curve, indicating substantial heterogeneity.

Nine studies with data on the accuracy of nasopharyngeal aspirate were included, providing 17 test comparisons, of which ten were included in the meta-analysis. All studies included children and were published between Nov 21, 1998, and May 3, 2021, with the number of sample sets ranging from 64 to 535 (median 150). Multiple index testing methodologies (non-automated culture [four reports], mycobacteria growth indicator tube [MGIT; one report], Xpert MTB/RIF [three reports], Xpert Ultra [one report], and PCR [one report]) were done. Microbiological reference tests induced sputum culture (six reports) and gastric aspirate culture (four reports; appendix pp 37–38). The proportion of children with a microbiologically confirmed diagnosis of pulmonary tuberculosis ranged from 3%^48^ to 38%.^43^ Model-estimated sensitivity was 65·2% (95% CI 52·0–76·4), specificity was 97·9% (96·0–99·0), DOR was 91·0 (37·8–218·8), PLR was 32·2 (15·8–66·1), and NLR was 0·35 (0·25–0·51; figure 2C). A meta-regression model using the different reference tests (sputum culture or gastric aspirate culture) as covariates showed a statistically significant difference in specificity but not sensitivity (appendix pp 38–40). The SROC curve showed some studies far from the curve, indicating heterogeneity (appendix p 48).

A second model of nasopharyngeal aspirate against composite reference standards (either microbiological or clinical diagnosis of pulmonary tuberculosis; appendix pp 40–42) with 12 comparisons gave an estimated sensitivity of 9·1% (95% CI 5·6–14·6), specificity of 99·9% (93·6–99·9), DOR of 168·4 (1·6–17959·1), PLR of 153·1 (1·4–16343·5), and NLR of 0·91 (0·87–0·95; figure 2D; appendix pp 40–42, 50).

18 studies, with 29 comparisons of oral swab samplings to microbiological reference standards, were identified. Of these, 20 (sampled between 2015 and 2022) were included in the meta-analysis. Ten used PCR for analysis, three used Xpert Ultra, two used culture, and one comparison each used Xpert MTB/RIF, tuberculosis loop-mediated isothermal amplification (LAMP), spoligotyping, auramine smear, and self-loading microfluidic (SLIM) assay. Eight comparisons had sputum culture as the reference standard, five used sputum culture and Xpert, two used sputum Xpert alone, four used gastric lavage culture and Xpert, and one used sputum or bronchiolar lavage culture (appendix pp 42–44). Seven comparisons included children as participants and 13 included adults as participants. Pooled sensitivity of oral swab samples was 56·7% (95% CI 44·3–68·2), specificity was 91·3% (81·0–96·3), DOR was 13·8 (5·6–34·0), PLR was 6·54 (3·0–14·5), and NLR was 0·47 (0·36–0·62; figure 2B). Meta-regression of oral sampling against reference standards classified as either sputum (16 reports) or gastric lavage (four studies)—allowing for unequal variances—identified a significant effect on specificity when sputum was the reference standard (appendix pp 45–46). The SROC curve (appendix p 51) indicated moderate heterogeneity.

We identified only a small number of studies of alternative sample types and meta-analysis was not done. These studies included mouthwash samples (three studies), saliva (four studies), oral cavity samples (two studies), and nasopharyngeal swabs (one study; appendix pp 46–47). An additional study on nasal swabs and one on saliva did not contain sufficient information to permit a summary of results.

**Figure 3:**
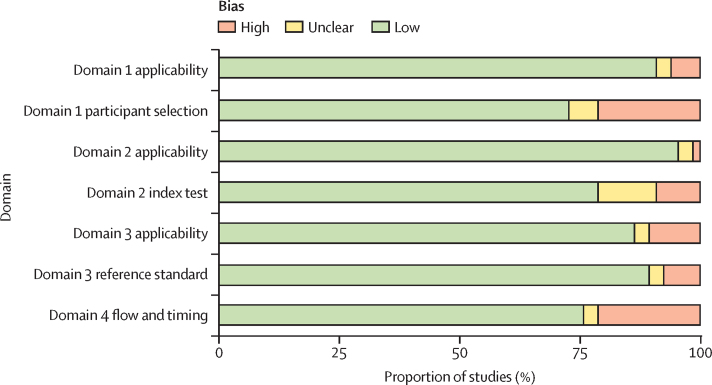
Proportion of studies with high, low, or unclear bias for each domain

For risk of bias assessment, studies performed before 1950 did not report on multiple QUADAS-2 domains, especially for participant selection and use of index tests (figure 3, appendix pp 52–53 for individual studies). In more recent studies there was a high risk of bias in domain 1 (participant selection) due to case-control designs and variability in reference standards used, with some reference standards less likely to correctly classify true tuberculosis status.

## Discussion

Diagnosing pulmonary tuberculosis is challenging and accurate sampling approaches that use easier-to-obtain specimens are urgently required. In this systematic review and meta-analysis, which included data on 24 899 sample comparisons from studies conducted over an 89-year period between 1933 and 2022, we found that three upper respiratory tract sample types show promise for accurately diagnosing pulmonary tuberculosis: laryngeal swabs with a pooled sensitivity of 57·8% and a specificity of 93·8%; nasopharyngeal aspirate with a pooled sensitivity of 65·2% and specificity of 97·9%; and oral swabs with a pooled sensitivity of 56·7% and specificity of 91·3%. These accuracy estimates are similar to currently approved tuberculosis diagnostic tools on non-sputum samples, such as urine lateral flow lipoarabinomannan assays in people with HIV^82^ and stool in children.^3^

Studies that used laryngeal swabs as a sample type were mostly from early 20th century Europe and gave a pooled sensitivity of 57·8% against microbiological reference standards. There was large variation in methods used over time as new medias and techniques were introduced and all were conducted before the advent of molecular diagnostics. These studies were mostly carried out among outpatients being investigated for tuberculosis who were unable to produce sputum, with the predominant reference standard being culture of gastric lavage. Use of laryngeal swab testing was discontinued with the advent of chemotherapy and the rapid decline of tuberculosis cases in northern Europe;^83^, ^84^ however, the original rationale for investigating this sample type (quick and well-tolerated outpatient test) reflects contemporary diagnostic priorities in high-burden countries today, as well as the requirements of the WHO Target Product Profile.^4^ Laryngeal swabs might hold promise as an alternative to expand access to tuberculosis diagnosis if re-evaluated using modern diagnostic platforms.

The second sample type reviewed was nasopharyngeal aspirate, which was mostly studied in children. Nasopharyngeal aspirate had a sensitivity of 65·5% compared with culture of gastric aspirate or induced sputum samples. However, given the low sensitivity of microbiological tests for tuberculosis in children,^85^ we also compared nasopharyngeal aspirate to a clinical reference standard, where sensitivity fell to 9%. This fall in sensitivity reflects the high proportion of children clinically diagnosed with tuberculosis in the absence of any positive microbiological test, a common occurrence in clinical practice that reflects the urgent need for improved diagnostic strategies. Nasopharyngeal aspirate and laryngeal swabs have the benefit of being able to be performed as an outpatient test, which could make them a viable alternative in settings where access to hospital services is limited due to cost, distance, and availability. Further research is needed to investigate whether sensitivity can be improved by optimising sample analysis, how nasopharyngeal aspirate performs in adults, and how nasopharyngeal aspirate could be used within diagnostic algorithms to increase microbiological diagnosis.^51^

Oral swabs gave a pooled sensitivity of 56·7% against microbiological reference standards. However, many of the studies were case-control studies with small numbers with a high risk of bias expected to overestimate sensitivity. There was also a wide variability in the swab types, sampling methodologies, and specimen analysis; nevertheless, oral swabs do offer an easy option for sample collection. Additional research is needed, including prospective diagnostic evaluations, to find standardised methodologies and assess accuracy in a wide range of populations, including children.

Limitations for this systematic review and meta-analysis include the variability of the methods of sample collection and analysis. We mitigated this variability by stratifying studies into groups of sample types for meta-analysis; however, within-group variability remained due to differences in index testing and reference standards used. The difference in reference standard, most commonly either sputum or gastric aspirate and lavage, and methods used for processing, either culture or Xpert, are sources of heterogeneity that can be seen in SROC curves. Modelling using meta-regression showed a statistically significant difference in specificity—but not sensitivity—between sputum and gastric lavage reference standards. This variability in study design highlights the difficulty in finding a reference standard in tuberculosis diagnosis and diagnostic accuracy studies as there is not one sample or analytic method that will guarantee the identification of tuberculosis disease correctly. By analysing reports by index type, we aimed to reduce variability and compare similar study designs from similar time periods with similar reference standards. However, differences in index test and reference standards microbiological test remained the largest source of heterogeneity in our analysis. For some studies from the early 20th century, we were unable to identify the full text despite extensive searches with librarians, especially if they were not published in English. Our inability to locate these studies may have biased results to studies in prominent journals that were more likely to be archived in libraries.

Although upper respiratory tract sampling seems a promising avenue for improving access to tuberculosis diagnostic testing, additional prospective studies are needed to optimise sampling and maximise sensitivity, including using novel technologies and techniques (such as mask testing for exhaled air). In children, where novel diagnostics are urgently needed, nasopharyngeal aspirate and potentially laryngeal swabs might offer alternative outpatient methodologies that can be used to widen access. Historical methods using laryngeal swabs showed similar sensitivity and higher specificity than modern studies using oral swabbing in much larger numbers of patients, some of whom were unable to expectorate. Updated evaluations of laryngeal swabbing are required to determine whether these findings are replicated in modern evaluations. Oral swabs are simple to collect and transport; however, this review has shown that more prospective study data are needed to understand whether sensitivity is sufficient for use in clinical practice. If sufficient, this method may give a sample type that could be used in outpatient settings for those unable to expectorate or in an inpatient setting for those too unwell to produce a sample. Overall upper respiratory tract sampling could offer an alternative non-invasive sample type that can be used to increase access to microbiological diagnosis for *M tuberculosis*.

## Data sharing

All study data and analytical code available at https://osf.io/9nuvq/.

## Declaration of interests

We declare no competing interests.
