# Supplementary material for: Accuracy of upper respiratory tract samples to diagnose Mycobacterium tuberculosis: a systematic review and meta-analysis
Source: Lancet Microbe. 2023 Oct;4(10):e811–21. doi: 10.1016/S2666-5247(23)00190-8 (PMC10547599; doi:10.1016/S2666-5247(23)00190-8)
Supplement: Supplementary appendix [file mmc1.pdf]

THE LANCET
Microbe

Supplementary appendix

This appendix formed part of the original submission and has been peer reviewed. We post it as supplied by the authors.

Supplement to: Savage HR, Rickman HM, Burke RM, et al. Accuracy of upper respiratory tract samples to diagnose *Mycobacterium tuberculosis*: a systematic review and meta-analysis. *Lancet Microbe* 2023; published online Sept 12. https://doi.org/10.1016/S2666-5247(23)00190-8.

Supplementary data tables and figures

Supplementary tables

S1: Search strategy

S2: Table to show definition of confirmed, probable or not PTB by study.

S3: Full study information

S4: Excluded studies

S5: Table to show study characteristics

S6: Table of data from laryngeal swab reports used in meta-analysis.

S7: Laryngeal swab reference standard meta-regression and table of results

S8: Table of data from naso-pharyngeal aspirate reports used in meta-analysis.

S9: Nasopharyngeal reference standard meta-regression and table of results

S10: Table of data from naso-pharyngeal aspirate reports and clinical diagnosis as a reference standard used in the meta-analysis.

S11: Data table to show analysis of reports of oral swabs as sample type.

S12: Oral swab reference standard meta-regression and table of results

S13: Data table to show analysis of reports of other sample type data.

Supplementary Figures:

SF1: Summary receiver operating curve of laryngeal sampling for active pulmonary tuberculosis, with random effects meta-analysis

SF2: Summary receiver operating curve of nasopharyngeal aspirate for active pulmonary tuberculosis, with random effects meta-analysis

SF3: Summary receiver operating curve of nasopharyngeal aspirate for active pulmonary tuberculosis when clinical diagnosis used as a reference standard, with random effects meta-analysis

SF4: Summary receiver operating curve of oral swab for active pulmonary tuberculosis, with random effects meta-analysis

SF5: Bias of individual studies presented via each domain question and overall rating.

SF6: Bias of individual studies presented by domain.

Table S1:

Search strategy for systematic review

Search terms
(“Mycobacterium tuberculosis” OR “Mycobacterium tuberculosis complex” OR “tuberculosis” OR “TB” OR “consumption” OR “wasting” OR “thysis” OR “pthysis OR “Koch’s Disease”)
AND
(“oral swab” OR “oral” OR “laryngeal swab” OR “laryngeal” OR “tonsils” OR “saliva” OR “Waldemeyers ring” OR “pharynx” OR “pharyngeal”)
AND
(“diagnosis” OR “diagnostics” OR “samples” OR “sampling”)
Plus database specific strategy terms
Medline (1847-1950): Human
Medline (1950 – current): Human, Clinical Key: Diagnosis
Global Health: As above, remove consumption
Global Health archive: As above, remove consumption

Supplementary table S2:

Table to show definition of confirmed, probable or not PTB by study.

Paper	Confirmed PTB	Number	Probable PTB	Number	Not TB	Number
Owens 2007	presence of a positive smear or culture for MTB		Probable PTB was suggested by hilar or mediastinal lymphadenopathy, local collapse/consolidation, severe bilateral but	94	No CXR changes as for probable TB not tested	0

	– taken from the probable TB group		asymmetric disease, cavitation or miliary changes. Chest radiographs were reviewed by JBSC who was blinded to the clinical details.			
Zar 2012	Any specimen positive for M. tuberculosis on culture	87	All others	255	Documented resolution of symptoms and signs at follow-up in children who didn't receive tuberculosis treatment	193
Zar 2013	Any induced sputum culture positive for M tuberculosis	30	All other children	167	Culture negative and documented resolution of symptoms and signs at a follow-up visit at 3 months in children who did not receive tuberculosis treatment	187
Cakir 2018		0	Diagnosis of TB disease was established in accordance with the World Health Organization's TB Standards case definition – respiratory symptoms, radiological findings, tuberculin skin test positivity, history of contact with active TB, and acid fast bacillus and MTB culture positivity	40		0
Zar 2019	Culture positive for Mycobacterium tuberculosis (excluding Ultra)	40	Culture negative, clinically diagnosed with TB	104	culture negative, not clinically diagnosed with TB, no tuberculosis treatment given, and documented improvement at 3-mo follow-up visit	51
Hanrahan 2018	Microbiologically using smear, culture or Xpert on any one of	4	No microbiological confirmation on any sample and at least two of the following: CXR consistent with TB, positive clinic response to	100	Unlikely TB were those Without mycobacteriological confirmation who did	15

	the samples collected		anti-TB treatment, documented exposure to TB or a positive TST.		not meet the criteria for unconfirmed TB.	
Osorio 2020			(i) signs/symptoms: (a) persistent cough (>2 weeks), unremitting cough; (b) weight loss/failure to thrive; © persistent (>1 week), unexplained fever reported by guardian; (d) persistent, unexplained lethargy or reduced playfulness; (e) infants 0–60 days with additional signs and symptoms like neonatal pneumonia, unexplained hepatosplenomegaly or sepsis-like illness; (ii) findings on chest X-ray congruent with pulmonary TB (presence of lymphadenopathy and/or abnormalities consistent with TB as new infiltrates) and read by two blinded operators (clinician and TB expert); (iii) history of exposure to M. tuberculosis within the preceding 12 months; or (iv) response to antituberculosis treatment yet no acid-fast bacillus on the sputum smear or a negative Xpert MTB/RIF test.	17	No TB	28
Song 2021			Participants had to have a visible cervical lymph node mass measuring >1 cm × 1 cm and persisting for >1 month despite antibiotic therapy for at least 5 days or a parenchymal abnormality on chest radiograph in addition to at least one of the following symptoms: 1) cough or wheezing >4 weeks not resolved after treatment with antibiotics, with cough continuing for at least 2 weeks after starting antibiotics (for hospitalized children only, respiratory distress or diagnosis of severe pneumonia not responding to antibiotics after 5	294		

			days or any cough >4 weeks despite at least 5 days of antibiotics), 2) moderate or severe malnutrition (defined as weight-for-height Z score <-2 standard deviations (SD) or -3SD, respectively) not responding after 3 weeks of treatment for malnutrition, and 3) reported fever >7 days not responding after 5 days of antibiotics or antimalarials. Children were excluded from the study if they were currently on tuberculosis treatment or isoniazid preventive therapy (IPT) or had received treatment for tuberculosis in the past year or IPT in the last 6 months.			

S Table S3:

Full descriptive data including methods used available as excel spreadsheet.

https://osf.io/9nuvq

Table S4:

Excluded studies with reasons

Title	Year	Journal	First Author	Exclusion reason
On Pulmonary Consumption, and on Bronchial and Laryngeal Disease, &c	1847	The Medico-chirurgical review		Full text unavailable
On Pulmonary Consumption; and on Bronchial and Laryngeal Disease; with Remarks on the Places of Residence Chiefly Resorted to by the Consumptive Invalid	1853	Edinburgh medical and surgical journal		Wrong study type - case report
The Pathology and Treatment of Pulmonary Tuberculosis; and on the Local Medication of	1854	Edinburgh medical and surgical journal		No relevant testing

Pharyngeal and Laryngeal Diseases, Mistaken for, or Associated with, Phthisis				
Contribution to the History of Laryngeal Phthisis	1875	Medico-chirurgical transactions	Marcet, W.	Background article
The Laryngeal Complications of Consumption	1882	British medical journal	Williamson, J. M.	No relevant testing
Phthisis of the Larynx	1883	Edinburgh medical journal	Mackenzie, G. Hunter	No relevant testing
Some Questions with Regard to Tuberculosis of the Upper Air Passages	1892	Transactions. Medico-Chirurgical Society of Edinburgh	M'Bride, P.	Wrong outcome
Clinical Remarks ON A CASE OF TUBERCULOUS DISEASE OF THE LUNGS AND LARYNX, SHOWING THE NEED FOR THE COMPULSORY NOTIFICATION OF PHTHISIS	1906	British medical journal	Bramwell, B.	wrong study design
Tuberculosis of the Larynx	1908	Proceedings of the Royal Society of Medicine	Hill, W.	Wrong study design - case report
The Throat and Nose in the Ã†tiology of Tuberculosis	1909	The Hospital		Background article
Tuberculosis of the Air-Passages above the Larynx	1910	The Hospital		Wrong outcome
Tuberculosis of the Tonsil, associated with Tuberculous Glands of Neck	1910	Proceedings of the Royal Society of Medicine	Carmichael, E. S.	Wrong outcome

Tuberculosis of Pharynx	1910	Proceedings of the Royal Society of Medicine	Wylie, A.	wrong study design
Chronic Tuberculosis of the Nose, Larynx, and Lungs	1911	Proceedings of the Royal Society of Medicine	Parker, C. A.	wrong study design
Laryngeal Tuberculosis	1912	Proceedings of the Royal Society of Medicine	Donelan, J.	wrong study design
Microscopical Sections from a Case of Tuberculous Ulcer of the Larynx; the First suggestive of Epithelioma, the Second of Non-bacillary Tuberculosis? Lupus	1912	Proceedings of the Royal Society of Medicine	Grant, J. D.	wrong study design
Some Observations on Tuberculosis of the Nose and Pharynx	1912	Bristol medico-chirurgical journal (1883)	Wright, A. J.	Wrong study design - case report
A Post-mortem Specimen of Laryngeal Tuberculosis	1913	Proceedings of the Royal Society of Medicine	Davis, E. D.	Wrong study design - case report
What Relation, if any, have the Faucial Tonsils to Pulmonary Tuberculosis?	1913	Transactions of the American Climatological Association for the year ... American Climatological Association	Ingals, E. F.	No relevant testing
Tuberculosis of Pharynx and Larynx	1913	Proceedings of the Royal Society of Medicine	McKenzie, D.	wrong study design

Some of the Problems of Private Sanatoria for Tuberculosis as observed during Ten Years' Experience in the Pottenger Sanatorium for Diseases of the Lungs and Throat	1914	Transactions of the American Climatological and Clinical Association. American Climatological and Clinical Association	Pottenger, F. M.	Background article
THREE YEARS' SANATORIUM EXPERIENCE OF LaRYNGEAL TUBERCULOSIS	1914	British medical journal	Thomson, S.	No relevant testing
Tuberculosis of the Larynx	1920	Transactions of the American Climatological and Clinical Association. American Climatological and Clinical Association	Forster, A. M.	Background article
Two Cases of Pulmonary Tuberculosis with Laryngeal Symptoms	1923	Proceedings of the Royal Society of Medicine	Franklin, P.	wrong study design
Treatment of Laryngeal Tuberculosis in Sanatoria	1923	Transactions of the American Climatological and Clinical Association. American Climatological	Parfitt, C. D.	Wrong outcome

		and Clinical Association		
Tuberculosis of the Larynx	1923	Proceedings of the Royal Society of Medicine	Renshaw, J. A.	wrong study design
TUBERCULOSIS OF THE LARYNX: Sir StClair Thomson's Report	1924	British medical journal		No relevant testing
Tuberculosis of the Larynx-Its Diagnosis and Treatment: A Plea for Closer Co-operation Between the Physician and Laryngologist	1924	Canadian Medical Association journal	Pentecost, R. S.	Background article
Tuberculosis of Nasal Bones	1924	Proceedings of the Royal Society of Medicine	Ridout, C. A.	wrong study design
The Mitchell Lecture ON TUBERCULOSIS OF THE LARYNX: ITS SIGNIFICANCE TO THE PHYSICIAN: Deliverd before the Royal College of Physicians of London, November 6th, 1924	1924	British medical journal	Thomson, S.	No relevant testing
Tuberculosis of Larynx	1927	Proceedings of the Royal Society of Medicine	Layton, T. B.	wrong study design
Infection of the Upper Respiratory Tract as an Etiological Factor in Pulmonary Disease	1930	Canadian Medical Association Journal	Smith, I. R.	Background article
Primary Infection and Pulmonary Tuberculosis in Adults. / Om Primaerinfektionen og Lungetuberkulosen hos Voksne	1931	Ugeskrift for Laeger	Heckscher, H.	Full text not available
Tuberculosis of Larynx	1931	Proceedings of the Royal Society of Medicine	Wylie, A.	wrong study design

INCIDENCE OF TUBERCULOSIS OF THE TONSIL	1932	Canadian Medical Association journal	Heaton, T. G.	Background article
Tubercle Bacilli in the Gastric Contents of Tuberculous Children. A Study of 59 Cases	1934	American Review of Tuberculosis and Pulmonary Diseases	Gourley, Ina	Full text not available
Present Concepts of Tuberculous Infection and Disease. Their Principles and Application	1935	American Review of Tuberculosis and Pulmonary Diseases	Opie, E. L.	Full text not available
Tuberculosis in Some Rare Situations, Namely Tonsils and Uterus	1936	The Indian medical gazette	Krishnaswamy, K. G.	Background article
The Importance of Gastric Lavage for the Demonstration of Tubercle Bacilli in Adults	1937	Acta Medica Scandinavica	Gullbring, A.	Wrong sample type
Tuberculosis of the Larynx	1939	Proceedings of the Royal Society of Medicine	Suggit, S.	wrong study design
The Routine Examination for Tubercle Bacilli in Sputum	1940	Tubercle	Hunter, R. A.	Wrong sample type
Gastric Contests in Tuberculous Children	1941	American Review of Tuberculosis and Pulmonary Diseases	Floyd, C	Wrong sample type
Observations on Tuberculosis of the Larynx	1941	Edinburgh medical journal	Martin, G. Ewart	Background article
Discussion on Primary Tuberculosis in Adolescents and Adults	1942	Proceedings of the Royal Society of Medicine	Kayne, G. G	Background article

The Clinical Value of Faeces Examination for Tubercle Bacilli in the Course of Pulmonary Tuberculosis. Results of a New Method of Examination. / Ueber den klinischen Wert des Stuhlnachweises von Tuberkelbazillen im Verlaufe der Lungentuberkulose. Ergebnisse eines neuen Untersuchungsverfahrens	1942	Deutsche Medizinische Wochenschrift	Wolf, J. E.	Wrong sample type
Tuberculosis of the Tonsils	1943	The American journal of pathology	Rather, L. J.	Background article
The Detection of Tubercle Bacilli by Deep Culture in Kirehner's Fluid Medium. / Der Nachweis der Tuberkelbazillen in der Tiefenkultur im flÃ¼ssigen NÃ¤hrboden nach Kirchner	1943	Zentralblatt fur Bakteriologie, Parasitenkunde, Infektionskrankheiten und Hygiene	Sula, L.	Wrong outcome - laboratory validation
Pulmonary Lavage in the Diagnosis of Tuberculosis. / O lavado pulmonar. No diagnÃ³stico etio-patogÃ©nico ou evolutivo da tuberculose	1944	Boletin de la Oficina Sanitaria Panamericana	De Abreu, M.	Wrong outcome
Tubercle Bacilli in the Stomach Contents of Healthy, Normal Adults exposed to Tuberculosis	1944	American Review of Tuberculosis and Pulmonary Diseases	Smith, C. R.	Wrong outcome
Tonsillectomy for tuberculosis	1945	Schweizerische Zeitschrift fur Tuberkulose. Revue suisse de la tuberculose. Rivista svizzera della tubercolosi	Escher, F.	Full text unavailable

About the oral tubercular primary complex and the post-primary lupoid of the gingiva	1945	Schweizerische medizinische Wochenschrift	Nager, : Fanconi	Full text unavailable
Tuberculosis of the tonsils	1946	Archives of otolaryngology	Bernstein, D.	Upper Resp TB
Laryngeal tuberculosis	1946	Diseases of the chest	Humphries, M. K., Jr.	Full text unavailable
Tuberculous ulcer of the tongue	1946	Prensa medica argentina	Steinberg, I. R.	Full text unavailable
Tuberculous sinuses	1946	The Medical journal of Australia	Stokes, E. H.	Full text unavailable
Pulmonary, laryngeal, and intestinal tuberculosis and pulmonary water cyst	1947	Gaceta medica de Lima		Full text unavailable
LARYNGEAL tuberculosis	1947	Manitoba medical review		Full text unavailable
Tuberculosis of the mouth	1947	Annals of dentistry	Brodsky, R. H.	Full text unavailable
On the functional pathology of laryngeal tuberculosis; comparative clinical and histological examinations	1947	Archiv fur Ohren-, Nasen- und Kehlkopfheilkunde	Eschweiler, H.	Full text unavailable
[Tuberculosis of the middle ear and larynx]	1947	Jornal do medico	Larroude, C.	Full text unavailable
Cutaneous and laryngeal tuberculosis and Charpy method	1947	Maroc medical	Lepinay	Upper Resp TB
Oral tuberculosis	1947	Revista dental de Chile	Louvel Bert, R.	Full text unavailable
Tuberculous tonsillitis	1947	Schweizerische medizinische Wochenschrift	Taillens, J. P.	Full text unavailable
Laryngeal tuberculosis in infancy	1947	Policlinico infantile	Tavani, E.	Full text unavailable

Tuberculosis of the oral mucosa	1948	Deutsche dentistische Zeitschrift	Bachmann, W.	Full text unavailable
Tuberculous laryngitis; a controlled study	1948	American review of tuberculosis	Black, J. P. M.	Full text unavailable
Comments on laryngeal tuberculosis	1948	L'union medicale du Canada	Brahy, J.	Full text unavailable
Tuberculosis of the larynx	1948	Acta oto-rhino-laryngologica Belgica	De Prest, R. A.	Full text unavailable
On tuberculosis of the nose, pharynx and tonsils	1948	Practica medica	Jimenez Encina, C.	Full text unavailable
Contribution to the study of the so-called latent tuberculosis of the lymphatic ring of Waldeyer	1948	Practica oto-rhino-laryngologica	Langraf, F.	Full text unavailable
Laryngeal tuberculosis; observations based on an experience of 28 years with laryngeal tuberculosis	1948	The Annals of otology, rhinology, and laryngology	Looper, E. A	Wrong outcome
Tuberculosis infection of the palatine tonsils	1948	Revista espanola de tuberculosis	Vassallo De Mumbert, A.	Full text unavailable
Tuberculosis of the tongue	1948	La Semana medica	Vergelin, H. E	Full text unavailable
Tonsil tuberculosis in childhood	1949	Schweizerische medizinische Wochenschrift	Banhidy, F.	Duplicate
Tuberculosis of the Tonsils in Infancy. / Tonsillentuberkulose im Kindesalter	1949	Schweizerische Medizinische Wochenschrift	BÃNhidy, F.	Wrong outcome
Tubercle Bacilli in Pathological Material from Patients with Miliary Tuberculosis : Study of	1949	Bulletin et Memoires de la	Bernard, E	Wrong sample type

114 Cases. / La recherche du bacille tuberculeux dans les tuberculoses micronodulaires. (Etude sur 114 cas.)		Societe Medicale des Hopitaux de Paris		
Nasal mucosal tuberculosis	1949	Nordisk medicin	Froste, N.	Full text unavailable
Tuberculosis of the Nasal Mucous Membranes. / NÃ¤sslemhinnetuberkulos	1949	Nordisk Medicin	Froste, N.	wrong study design
Tuberculous larynx	1949	Archivos medicos mexicanos	Fumagallo Perez, L.	Full text unavailable
Tuberculosis of the tongue	1949	Dental items of interest	Gergely, L	Full text unavailable
Laryngeal tuberculosis	1949	Archives of otolaryngology	Looper, E. A.	Wrong outcome
[Tuberculosis of the larynx]	1950	Medicina, cirurgia, farmacia	Blundi, E	Full text unavailable
[Laryngeal tuberculosis]	1950	Acta oto-rhino-laryngologica Belgica	Dupont, P.	Full text unavailable
[Statistical data on the frequency and evolution of laryngeal tuberculosis before the use of streptomycin]	1950	Journal de medecine de Lyon	Piaget, F	Wrong outcome
Failure in the Demonstration of Tubercle Bacilli in Gastric Washings. / Svikt i pÃ¡visingen av tuberkelbasiller i ventrikkelskyllevann	1951	Nordisk Medicin	Rambl, K.	Wrong sample type
A Nonchromogenic Culture of an Acid-Fast Bacillus isolated from the Nasal Mucus of a Leprosy Patient; its Virulence for Laboratory Animals	1952	International Journal of Leprosy	De Souza-Araujo, H. C.	Wrong outcome
Tuberculosis in Childhood, as disclosed or confirmed by Cultivation of Mycobacterium tuberculosis from Gastric Content	1952	Medical Journal of Australia	Webster, R.	Wrong sample type

Unclassified Mycobacteria in the Gastric Contents of Healthy Personnel and of Patients of a Tuberculosis Hospital	1960	American Review of Respiratory Disease	Atwell, R. J	Wrong sample type
Pulmonary tuberculosis in dog diagnosed by a positive culture of Mycobacterium tuberculosis from laryngeal swab	1967	American Review of Respiratory Disease	Trujillo-Rojas, R. A.	No relevant testing
LARYNGEAL TUBERCULOSIS	1977	American Journal of Roentgenology	Lindell, M. M	Wrong outcome
Diagnosis of nasopharyngeal tuberculosis by detection of tuberculostearic acid in formalin fixed, paraffin wax embedded tissue biopsy specimens	1988	Journal of clinical pathology	Arnold, M	Wrong outcome
LARYNGEAL TUBERCULOSIS REVISITED	1992	American Family Physician	Riley, E. C.	Full text unavailable
An exceptional localization of upper respiratory tract tuberculosis: The tonsil	1996	Semaine Des Hopitaux	Raji, A	Wrong study design - case report
Risk of tuberculosis transmission in dentistry	1997	AAOHN Journal	Murphy, D. C	No relevant testing
Search for Mycobacterium paratuberculosis DNA in orofacial granulomatosis and oral Crohn's disease tissue by polymerase chain reaction	1997	Gut	Riggio, M. P	Wrong pathogen
Diagnosis of tuberculosis in sputum negative patients in Dar es Salaam	1999	East African Medical Journal	Aris, E. A	Full text unavailable
Changing trends in clinical manifestations of laryngeal tuberculosis	2000	Laryngoscope	Shin, J. E	Wrong outcome
Laryngeal tuberculosis	2000	American Journal of Otolaryngology	Yencha, M. W	Wrong outcome
Laryngeal tuberculosis	2004	Lancet Infectious Diseases	Krecicki, T	wrong study design

A comparative study of the diagnosis of pulmonary tuberculosis using conventional tools and polymerase chain reaction	2006	Indian Journal of Tuberculosis	Kavita, Modi-Parekh	Wrong sample type
Tuberculosis of tonsil associated with pulmonary foci	2008	Indian Journal of Otolaryngology and Head & Neck Surgery	Santosh, U. P	wrong study design
Laryngeal involvement in patients with active pulmonary tuberculosis	2008	European Archives of Oto-Rhino-Laryngology	Topak, M	Upper Resp TB
Is routine pathological examination required in South African children undergoing adenotonsillectomy?	2009	SAMJ - South African Medical Journal	Lierop, A. C. van	Background article
Laryngeal tuberculosis	2010	Journal of Otolaryngology-Head & Neck Surgery	Cherkaoui, A	wrong study design
Clinical utility of a commercial LAM-ELISA assay for TB diagnosis in HIV-infected patients using urine and sputum samples	2010	PloS one	Dheda, Keertan	Wrong outcome
18F-FDG PET/CT findings of pharyngeal tuberculosis	2010	Annals of Nuclear Medicine	Ito, K.	Background article
Tuberculosis of the oral cavity: a systematic review	2010	European Journal of Oral Sciences	Kakisi, O. K.	Background article
The TDR tuberculosis specimen bank: a resource for diagnostic test developers	2010	International Journal of Tuberculosis and Lung Disease	Nathanson, C. M.	Background article

Nasal tuberculosis--an update of current clinical and laboratory investigation	2011	The Journal of laryngology and otology	Masterson, L.	wrong study design
Detection and identification of Mycobacterium tuberculosis and Mycobacterium bovis from clinical species using DNA microarrays	2012	Journal of Veterinary Diagnostic Investigation	Jia, K	Wrong population
Laryngoscopic characteristics and diagnosis of laryngeal tuberculosis	2012	Journal of Otolaryngology and Ophthalmology of Shandong University	Yu, Ping	wrong study design
Assessment of the Xpert MTB/RIF assay for diagnosis of tuberculosis with gastric lavage aspirates in children in sub-Saharan Africa: a prospective descriptive study	2013	The Lancet. Infectious diseases	Bates, Matthew	Wrong sample type
Secondary laryngeal tuberculosis revisited	2015	Lung India	Lodha, J. V	wrong study design
Morphological aspects in tuberculosis of oral cavity our experience and a review of the literature attempt	2015	Romanian Journal of Morphology and Embryology	Popescu, M. R	wrong study design
Tuberculous cheilitis revealing pulmonary tuberculosis. / ChÃ©ilite tuberculeuse rÃ©vÃ©lant une tuberculose pulmonaire	2016	Pan African Medical Journal	Bricha, M	Upper Resp TB
Tuberculous cheilitis revealing pulmonary tuberculosis	2016	Pan African Medical Journal	Bricha, M	Duplicate
Respiratory microbes present in the nasopharynx of children hospitalised with suspected pulmonary tuberculosis in Cape Town, South Africa	2016	BMC Infectious Diseases	Dube, Felix S	No relevant testing

Nasal swab real-time PCR is not suitable for in vivo diagnosis of bovine tuberculosis	2017	Pesquisa VeterinÃ¡ria Brasileira	Mayer, F. Q	Wrong population
ORAL LOCALIZATION OF TUBERCULOSIS: CASE REPORT AND LITERATURE REVIEW	2017	Journal of Medical and Surgical Research	Nabih, O	wrong study design
Performance of the Xpert MTB/RIF assay in the diagnosis of tuberculosis in formalin-fixed, paraffin-embedded tissues	2017	International journal of mycobacteriology	Polepole, Pascal	Wrong sample type
Repertoire of bacterial species cultured from the human oral cavity and respiratory tract	2018	Future Microbiology	Fonkou, M. D. M	wrong study design
GeneXpert MTB/RIF Outperforms Mycobacterial Culture in Detecting Mycobacterium tuberculosis from Salivary Sputum	2018	BioMed Research International	Shi, Jin	Wrong sample type
Detection of Mycobacterium tuberculosis purified ESAT-6 (Rv3875) by magnetic bead-coupled gold nanoparticle-based immuno-PCR assay	2018	International Journal of Nanomedicine	Singh, N	Wrong outcome - lab validation
Laryngeal tuberculosis diagnosed in a pathological laboratory in Senegal (2011-2015). / La tuberculose laryngÃ©e diagnostiquÃ©e dans un laboratoire d'anatomie pathologique du sÃ©nÃ©gal (2011-2015)	2018	Bulletin de la SociÃ©tÃ© de Pathologie Exotique	Thiam, I	wrong study design
Molecular Detection of Mycobacterium tuberculosis from Stools in Young Children by Use of a Novel Centrifugation-Free Processing Method	2018	Journal of clinical microbiology	Walters, Elisabetta	Wrong sample type

Guidance for Studies Evaluating the Accuracy of Biomarker-Based Nonsputum Tests to Diagnose Tuberculosis	2019	The Journal of infectious diseases	Drain, Paul K	Background article
Lipoarabinomannan in sputum to detect bacterial load and treatment response in patients with pulmonary tuberculosis: Analytic validation and evaluation in two cohorts	2019	PLoS medicine	Kawasaki, Masanori	Wrong sample type
Urine Xpert MTB/RIF for the diagnosis of childhood tuberculosis	2019	International Journal of Infectious Diseases	Lopez, A. L	Wrong outcome
Liquid mycobacterial culture outcomes after different sputum collection techniques before and during treatment	2019	Tuberculosis (Edinburgh, Scotland)	Lourens, Madeleine	Wrong sample type
Prevalence of oral lesions in tuberculosis: a cross sectional study	2019	Journal of Family Medicine and Primary Care	Purnendu, Rout	wrong study design
Sample adequacy controls for infectious disease diagnosis by oral swabbing	2020	PloS one	Deviaene, Meagan	Wrong outcome - lab validation
Tonsillar Tuberculosis	1945	Fichero medico terapeutico	Hamuy, D. J	Full text unavailable
[Examination of stomach contents and laryngeal mucus in adult tubercular patients]	1945	Problemy tuberkuleza	Klebanova, A. A.	Full text unavailable
Laryngeal tuberculosis	1946	Archives of otolaryngology	Auerbach, O.	Background article
Tonsils and tuberculosis	1946	Medecine et hygiene	Vetter, H.	Full text unavailable
Subcutaneous gum tuberculosis and pulmonary tuberculosis	1947	Le Poumon	Delord, M.	Full text unavailable

Tonsils and tuberculosis	1947	Schweizerische medizinische Wochenschrift	Vetter, H.	Full text unavailable
About the development of tonsil tuberculosis	1947	Schweizerische Zeitschrift fur Pathologie und Bakteriologie. Revue suisse de pathologie et de bacteriologie	Wegelin, C.	Full text unavailable
Histological research on the participation of tonsillary lymphatic tissue in the tubercular process	1948	Annali dell'Istituto "Carlo Forlanini"	Colantuono, P.	Full text unavailable
Tomography of larynx in disseminated pulmonary tuberculosis	1949	Diseases of the chest	Espinoza Galarza, M.	Full text unavailable
Hypopharyngeal (Laryngeal) Swabbing for the Cultural Diagnosis of Pulmonary Tuberculosis. A Statement of the Laboratory Subcommittee	1956	American Review of Tuberculosis and Pulmonary Diseases	A. J. O'Hea.	Background article
Catarrhal Affections of the Nasal Passages as a Cause of Pulmonary Phthisis, with Special Reference to the Question of Heredity	1886	Transactions of the ... Annual Meeting of the American Climatological Association. American Climatological Association. Annual Meeting	Jarvis, W. C.	Wrong outcome
The Tonsil in Tuberculosis	1898	The Hospital		Wrong test type
The Recognition of Early Changes in the Larynx in Tuberculosis	1913	Transactions of the American	Casselberry, W. E.	Wrong test type

		Climatological Association for the year ... American Climatological Association		
Laryngeal Lesion associated with Apparent Miliary Tuberculosis of the Lung	1927	Proceedings of the Royal Society of Medicine	Howarth, W.	Wrong study design - case report
TUBERCULOSIS OF THE LARYNX	1929	British medical journal	Howarth, W. G.	Background article
TUBERCULOSIS OF THE LARYNX	1929	British medical journal	Thomson, S.	Background article
Diagnostic Value of Direct and Cultural Examination of Laryngeal Secretion in Tuberculosis. / Importanza diagnostica della ricerca dei bacilli di Koch mediante l'esame diretto e culturale del secreto prelevato in laringe	1936	Rivista di Patologia e Clinica della Tuberculosi	Bernabo-Silorata, A	Full text unavailable
Negative Sputum and Pulmonary Tuberculosis	1937	Tubercle	Davies, G. I.	wrong study design
The Value of Exact Sputum Examination (Laryngeal Swab Culture) in the Diagnosis and Management of Pulmonary Tuberculosis	1943	Tubercle	Munro-Ashman, D	Wrong study design - case report
The incidence, treatment and prognosis of tuberculous laryngitis in pulmonary tuberculosis	1947	The Medical press	Lambert, V.	Full text unavailable
Relationship between pulmonary tuberculosis and tonsils	1948	Minerva medica	Puricelli, P. J.	Full text unavailable
Contribution to the study of so-called latent tuberculosis of the Waldeyer's lymph ring	1949	Schweizerische medizinische Wochenschrift	Langraf, F.	Full text unavailable

Tuberculous laryngitis and tracheo bronchitis	1949	The Annals of otology, rhinology, and laryngology	O'Keefe, J. J.	Full text unavailable
Cervical lymph node tuberculosis and the tonsils	1949	Acta pathologica et microbiologica Scandinavica	Pentti, M.	Background article
Tonsillar swab culture in lung tuberculosis	1950	Acta tuberculosea Scandinavica	Adler, H	Full text unavailable
[Role of the nasopharynx complex in primary tuberculosis]	1950	Revue de la tuberculose	Couve, P	Full text unavailable
[Comparative bacteriologic research on secretion withdrawn from the larynx in pulmonary tuberculosis]	1950	Archivio italiano di otologia, rinologia e laringologia	Redoglia, F.	Full text unavailable
An investigation of the occurrence of fungi in 250 laryngeal swabs from tuberculous patients	1953	Acta Path. et Microb. Scandinavica	Reiersol, S.	Wrong outcome
Direct Trachal Lavage for Rapid Recovery of Mycobacterium tuberculosis from the Respiratory Tract	1956	Brit. J. Tuberculosis	Jones, J. S.	Wrong outcome
The Laryngeal Swab Method of diagnosing Pulmonary Tuberculosis	1956	Monthly Bull. Ministry of Health & Pub. Health Lab. Service (directed by Med. Res. Council)	Thomas, C. H. H.	Wrong test type
Isolation of mycobacteria from tonsils, naso-pharyngeal secretions and lymph nodes in East Anglia	1970	Tubercle	Stewart C, J	Epidemiological study

Sensitivity and specificity of PCR for detection of Mycobacterium tuberculosis: a blind comparison study among seven laboratories	1994	Journal of clinical microbiology	Noordhoek, G. T	Wrong outcome - lab validation
Identification of novel tuberculosis diagnostic biomarkers in plasma and saliva	2016	European Respiratory Journal	Jacobs, R	Wrong test type biomarkers
Suitability of saliva for Tuberculosis diagnosis: comparing with serum	2017	BMC Infectious Diseases	Namuganga, Anna Ritah	Wrong outcome - lab validation
An optimised saliva collection method to produce high-yield, high-quality RNA for translational research	2020	Plos One	Sullivan, R	Wrong outcome
Primary tuberculosis cutis orificialis; a different face of the same coin	2021	Idcases	Ali, G. A.	Wrong study type - case report
Next-Generation Digital Biomarkers for Tuberculosis and Antibiotic Stewardship: Perspective on Novel Molecular Digital Biomarkers in Sweat, Saliva, and Exhaled Breath	2021	Journal of medical Internet research	Brasier, Noe	Biomarker study - wrong test not micro
Primary tonsillar tuberculosis	2021	Klimik Dergisi	DaÅŸli, S	Wrong study type - case report
Tuberculosis in the head and neck: changing trends and age-related patterns	2021	Laryngoscope	Gehrke, T.	Wrong study type - case report
New developments in tuberculosis diagnosis and treatment	2022	Breathe	Gil, C. M.	Wrong study type - review
Oro-facial tuberculosis - Is it still an enigmatic entity?	2021	Indian Journal of Pathology and Microbiology	Gupta, L.	Wrong study type - case report
A rare association of tonsillar tuberculosis and lichen scrofulosorum	2021	Egyptian Journal of Otolaryngology	Harit, A.	Wrong study type - case report

Dysphagia as the Presenting Symptom of Laryngeal Tuberculosis	2021	Cureus	Kandah, E.	Wrong study type - case report
Host-Based Biomarkers in Saliva for the Diagnosis of Pulmonary Tuberculosis in Children: A Mini-Review	2021	Frontiers in Pediatrics	Khambati, N	Biomarker study - wrong test not micro
A narrative review of exploring potential salivary biomarkers in respiratory diseases: still on its way	2021	Journal of Thoracic Disease	Li, C. X.	Biomarker study - wrong test not micro
Cervical Tuberculosis Combined With Papillary Thyroid Carcinoma With Lateral Neck Metastasis		Ent-Ear Nose & Throat Journal	Lin, H. Y.	Wrong study type - case report
The history of tuberculosis of the larynx	2021	Laryngo-Rhino-Otologie	Luckhaupt, H.	Wrong study type - review
Suspected IgG4-related disease of the submandibular salivary gland and periorbital soft tissue in a man with latent tuberculosis	2021	Bmj Case Reports	McGreal-Bellone, A.	Wrong study type - case report
Primary tonsillar tuberculosis in a pediatric patient case report and literature review	2021	Medicine (Baltimore)	Moisa, S. M.	Wrong study type - case report
Identification of novel salivary candidate protein biomarkers for tuberculosis diagnosis: a preliminary biomarker discovery study	2021	Tuberculosis	Mutavhatsindi, H.	Biomarker study - wrong test not micro
Determining the prevalence of rifampicin resistant tuberculosis in a tertiary care centre of north India by using rapid culture method and gene Xpert	2021	Journal of Pure and Applied Microbiology	Pokhriyal, B. C	Wrong sample type - not upper respiratory tract
Laryngeal tuberculosis: a neglected diagnosis	2022	Bmj Case Reports	Raj, R.	Wrong study type - case report
Lingual primary tuberculosis mimicking malignancy	2021	Annals of Medicine and Surgery	Razem, B.	Wrong study type - case report
Primary tuberculosis of the pyriform sinus: A case report	2022	Annals of Medicine and Surgery	Touihmi, S.	Wrong study type - case report

An Analysis of Xpert Test for Diagnosing Maxillofacial Tuberculosis		Journal of Maxillofacial & Oral Surgery	Tripathi, R.	Wrong outcome - diagnosing URT TB
A look inside: oral sampling for detection of non-oral infectious diseases	2021	Journal of Clinical Microbiology	Valinetz, E. D.	Wrong study type - review
Life-Threatening Stridor due to Laryngeal Tuberculosis in the COVID-19 Era: Report of a Case		Ent-Ear Nose & Throat Journal	Valjarevic, S.	Wrong study type - case report
Two cases of tuberculous retropharyngeal abscess in adults	2021	The Journal of international medical research	Xu, Xiaofeng	Wrong study type - case report
Case Report: Lingual Tuberculosis Reveaed by a Cold Abcess		Indian Journal of Otolaryngology and Head & Neck Surgery	Younes, H	Wrong study type - case report
Laryngeal and voice disorders in patients with pulmonary tuberculosis	2021	Iranian Journal of Otorhinolaryngology	Youssef, G	Wrong outcome - diagnosing URT TB
The participation of tonsils in childhood tuberculosis	1947	Revista chilena de pediatria	Pena Cereceda, J	Full text unavailable
A Note on the Laryngeal Mirror Test for the Detection of Tubercle Bacilli	1936	Tubercle	Wood, W. B.	Wrong study design - case report
Tuberculosis, of Tonsils: Its Relation in Children with Tubercle Bacilli in Gastric Contents	1941	American Review of Tuberculosis and Pulmonary Diseases	Rosencrantz, E.	Wrong test type histology
On the occurrence and treatment of laryngeal tuberculosis in patients at the pulmonary clinic	1945	Duodecim; laaketieteellinen aikakauskirja	Santalarti, V.	Background article

[Gastric lavage and laryngeal swab in search of Koch's bacillus]	1949	Revista brasileira de medicina	Silva Junior, E.	Review article
[Laryngeal tuberculosis associated with pulmonary tuberculosis; its incidence, prognosis and treatment]	1950	The Medical press		Wrong outcome - URT TB
Proteomics in Biomarker Discovery for Tuberculosis: Current Status and Future Perspectives	2022	FRONTIERS IN MICROBIOLOGY	Guo, JB	Wrong test type - biomarkers
Improved Conventional and New Approaches in the Diagnosis of Tuberculosis	2022	FRONTIERS IN MICROBIOLOGY	Dong, BY	Review article
Performance of Xpert Ultra nasopharyngeal swab for identification of tuberculosis deaths in northern Tanzania	2022	Clinical microbiology and Infection	Costales, C.	Wrong population – post-mortem

Supplementary Table S5: Study characteristics

**Year**	**Author**	**Country**	**Study design**	**Setting**	**Technique and Device**	**Site of sampling**	**Participants**	**Index test analysis**	**Reference test**	**Included in analysis**	**Male**	**HIV positive (if study after 1981)**

**Laryngeal swab**											
1933	Kereszturi (1)	USA	Diagnostics evaluation	Hospital	Throat swab	Children with TB	Culture	Gastric lavage culture	10	NR	
1937	Allodi (2)	Italy	Diagnostics evaluation	Clinic	Nichrome swab passed to larynx with 'fenestrated' cannula using a mirror.	Clinic patients	Culture	Gastric lavage culture	40	NR	
1941	Nassau (3)	UK	Diagnostics evaluation	Sanatorium	Sterilised angulated swab with cotton wool, passed to larynx and cough.	Discharged 1940 – unable to expectorate	Culture	Sputum culture culture	166	NR	
1948	Forbes (4)	UK	Diagnostics evaluation	Tuberculosis Hospital	Sterilised angulated nichrome swab with cotton wool, passed to larynx and cough.	Inpatient sputum culture negative	Culture	Gastric lavage culture	100	100	
1948	Hounslow (5)	UK	Diagnostics evaluation	Tuberculosis Hospital	Sterilised angulated nichrome swab with cotton wool, passed to larynx and cough.	Hospital patients – unable to expectorate	Culture	Gastric lavage culture	193	NR	
1950	Duggan(6)	Canada	Diagnostics evaluation	Sanatorium	Sterilised angulated swab with cotton wool, passed to larynx and cough.	Inpatients and Outpatients both adults and children	Culture	Gastric lavage culture	100	NR	
1950	Renoux(7)	France	Diagnostics evaluation	Sanatorium	Sterilised angulated nichrome swab with cotton wool, passed to larynx and cough.	Sanatorium patients – sputum negative	Culture	Gastric lavage culture	56	NR	
1951	Armstrong (8)	Canada	Diagnostics evaluation	Hospital	Sterilised angulated swab with cotton wool, passed to larynx and cough.	Hospital patients on TB treatment	Culture	Gastric lavage culture	1024	NR	
1951	Szabo(9)	Hungary	Diagnostics evaluation	Not stated	Not described	Smear negative TB patients	Culture	NR	2174	NR	
1953	Chaves (10)	USA	Diagnostics evaluation	Chest clinic	Sterilised angulated nichrome swab with cotton wool, passed to larynx and cough.	Clinic patients presumptive TB	Culture	Gastric lavage culture	1418	NR	
1954	Frostad (11)	Norway	Diagnostics evaluation	TB Hospital	Not described	TB hospital patients	Culture	Gastric lavage culture	1500	NR	
1955	Campbell (12)	Australia	Diagnostics evaluation	Chest clinic	Sterilised angulated swab with cotton wool, passed to larynx and cough.	Chest clinic – unable to expectorate	Culture	Sputum culture	578	NR	
1955	Lees (13)	UK	Diagnostics evaluation	Mass screening/asymptomatic	Not described	Mass screening admitted for swabs	Culture	Bronchial lavage culture	144	NR	

1955	Lind (14)	Sweden	Diagnostics evaluation	Not stated	Stainless wire with calcium alginate advanced to glottis and cough	Patients with lesions on XR	Culture	Gastric lavage culture	121	NR	
1955	Wallace(15)	UK	Diagnostics evaluation	Hospital	Either cotton wool AC or alginate BDE	Inpatients on TB treatment	Culture	Sputum culture	163	NR	
1956	Edwards (16)	English	Diagnostics evaluation	Chest clinic	Not described	Outpatients – presumptive TB	Culture	NR	1019	NR	
1956	Engbaek (17)	Denmark	Diagnostics evaluation	Hospital	Sterilised angulated swab with cotton wool, passed to larynx and cough.	Outpatients – unable to expectorate	Culture	Gastric lavage culture	1231	NR	
1956	Tonge (18)	Australia	Diagnostics evaluation	Chest clinic	Sterilised angulated swab with cotton wool, passed to larynx and cough.	Outpatients – presumed and known TB, unable to expectorate	Culture	Gastric lavage culture	465	NR	
1962	Hsing (19)	Taiwan	Diagnostics evaluation	Hospital	Sterilised angulated swab with cotton wool, passed to larynx and cough.	Hospital patients	Culture	Gastric lavage culture	1320	NR	
1962	Velu (20)	India	Diagnostics evaluation	Outpatient treatment study	Sterilised angulated swab with cotton wool, passed to larynx and cough.	Outpatients – bacteriologically confirmed TB	Culture	Sputum culture	2809	NR	
1962	Kertay(21)	Germany	Diagnostics evaluation	State TB institute	Not described	TB hospital patients treated for TB	Culture	Sputum culture	212	NR	
1965	Hauge (22)	Norway	Diagnostics evaluation	Hospital	Not described	Hospital patients – PTB on XR and clinical	Culture	NR	157	NR	
1966	Pechacek (23)	Czech republic	Diagnostics evaluation	TB hospital	Not described	TB hospital patients – referred as PTB some already treated	Culture	Sputum culture	525	NR	
1968	Lloyd (24)	English	Diagnostics evaluation	Hospital	Stainless steel wire, wet cotton wool, bent to angle	Paediatric inpatients with PTB	Culture	Gastric lavage culture	60	NR	
1970	Mankiewicz (25)	Canada	Diagnostics evaluation	Screening/Contacts/Asymptomatic	Alginate wool sterilised angulated swab, into larynx and cough	Outpatients – unable to expectorate	Culture	NR	1199	NR	
1999	Thakur (26)	India	Diagnostics evaluation	Hospital outpatients	Sterilised angulated swab, passed to larynx and cough.	Outpatients – children under 14 probable TB	Culture	NR	51	33	
1948	Gilje(27)	Norway	Diagnostics evaluation	Sanitorium	Sterilised angulated swab with cotton wool, passed to larynx and cough (Laryngeal streak)	Inpatients – unable to expectorate	Culture	Gastric lavage culture	400	NR	

1950	Lundar (28)	Norway	Diagnostics evaluation	Sanitorium	Sterilised angulated swab with cotton wool, passed to larynx and cough (Laryngeal streak)		Inpatients – unable to expectorate	Culture	Gastric lavage culture	304	NR	
1951	Gilje(29)	Norway	Diagnostics evaluation	Sanitorium	Sterilised angulated swab with cotton wool, passed to larynx and cough (Laryngeal streak)		Inpatients – unable to expectorate	Culture	Gastric lavage culture	863	NR	
1952	Roald (30)	Norway	Diagnostics evaluation	Sanitorium	Sterilised angulated swab with cotton wool, passed to larynx and cough (Laryngeal streak)		Inpatients	Culture	Gastric lavage culture	483	NR	
1952	Smedsrud (31)	Norway	Diagnostics evaluation	Sanitorium	Sterilised angulated swab with cotton wool, passed to larynx and cough (Laryngeal streak)		Inpatients	Culture	Sputum culture	71	NR	
1953	Laes (32)	Finland	Diagnostics evaluation	Sanitorium	Sterilised angulated swab with cotton wool, passed to larynx and cough (Laryngeal streak)		Inpatients – negative Ziehl-Nielson	Culture	Gastric lavage culture	232	NR	
Nasopharyngeal aspirate												
1998	Franchi (33)	Peru	Diagnostic evaluation	Hospital	Feeding catheter	Oropharynx	Children presumptive TB	Culture/PCR	Gastric aspirate culture	64	NR	0
2007	Owens (34)	Uganda	Diagnostic evaluation	Hospital	Graduated suction catheter	Oropharynx	Children presumptive TB	Culture	Induced sputum culture	94	57	44
2018	Cakir (35)	Turkey	Diagnostic evaluation	Hospital	Suction catheter	Oropharynx	Children presumptive TB	Culture	Gastric aspirate culture	40	18	NR
2012	Zar (36)	South Africa	Diagnostic evaluation	Hospital	Sterile catheter with mucus trap	Nasopharynx	Children presumptive TB	Culture/Xpert MTB/RIF	Induced sputum culture	535	NR	117
2013	Zar (37)	South Africa	Diagnostic evaluation	Primary care clinic	Sterile catheter with mucus trap	Nasopharynx	Children presumptive TB	Xpert MTB/RIF	Induced sputum culture	384	181	31
2018	Hanrahan (38)	South Africa	Diagnostic evaluation	Primary care clinic	Fasted 2 hours sterile catheter and mucus trap	Nasopharynx	Children presumptive TB	Culture/Xpert	Induced sputum culture	105	63	21

								MTB/ RIF				
2019	Zar (39)	South Africa	Diagnostic evaluation	Hospital	Sterile catheter with mucus trap	Nasopharynx	Hospitalised presumptive PTB	Xpert Ultra	Induce d sputum culture	195	NR	32
2021	Song (40)	Kenya	Cross sectional	Inpatient and Outpatient	French tubing and mucus trap	Nasopharynx	Children presumptive TB	Xpert MTB/ RIF / MGIT	Gastric aspirat e MGIT	294	149	73
2020	Osorio (41)	Mozambique	Retrospective cross-sectional	Hospital	Not stated	Nasopharynx	Children with severe acute malnutrition	Xpert MTB/ RIF / MGIT	NR	45	24	10
2009	Al-Aghbari (42)	Yemen	Cohort	Hospital	Aspiration via a mucus trap connected to a suction device	Nasopharynx	Children suspected TB	Cultur e	n/a	213	NR	NR
Oral swab												
2014	Wood (43)	South East Asia	Conference abstract	NR	NR	NR	NR	NR	NR	54	NR	NR
2015	Wood (44)	South Africa	Case control	TB clinic	Omniswab	Inside of cheek 10 seconds	TB clinic patients	PCR	Sputu m Xpert MTB/ RIF	20	8	0
2019	Luabeya (45)	South Africa	Case control	TB Clinic	Omniswab x 3, Puritan Purflock Ultra x 1 cheek	Tongue swabs	TB clinic patients	PCR	Sputu m Xpert MTB/ RIF	130	N R	NR
2019	Mesman (46)	Peru	Case control	TB clinic	Omniswab in lysis buffer, Omniswab in PBS, FTA card	Cheek	TB clinic patients	Xpert MTB/ RIF	Sputu m MGIT	33	NR	NR
2019	Nicol (47)	South Africa	Prospective diagnostic evaluation	Hospital	Omniswab/Purita n Purflock	Cheek (right and left)	Presumptive pulmonary TB	PCR	Induce d sputum Xpert MTB/ RIF	165	78	18

2020	Flores (48)	Peru	Prospective diagnostic evaluation	Not stated	Omniswab in sterile lysis buffer or EasiCollect FTA card	Inside of both cheeks 10 seconds	Presumptive pulmonary TB	PCR	Bacteriologically confirmed	288	NR	0
2020	Mesman (49)	Peru	Case control	Health centres	Omniswab, Easicollect FTA card	Inside of each cheek 10 seconds	Culture confirmed TB	PCR	Sputum culture	123	82	4
2020	Molina-Moya (50)	Moldova	Cross sectional	Hospital	Primeswab	Swab cheek 7-8 seconds	Clinical diagnosis presumptive TB	PCR	Sputum culture	266	157	NR
2020	Lima (51)	Brazil	Case control	Prison	Not stated	Tongue 10 seconds	Sputum Xpert positive	Xpert Ultra	Sputum Xpert Ultra	128	NR	NR
2021	Ealand (52)	South Africa	Case control	Hospital	Copan swab	Tongue 6-8 seconds	Young children less than equal 5 clinically diagnosed with TB	Culture, Auramine/spogliotyping.MTBC DNA	Gastric aspirate culture /Xpert	35	25	6
2021	Song (53)	China	Prospective diagnostic evaluation	TB Hospital	Swab	Dorsum tongue 7-8 times	> 16 years symptoms suggestive pulmonary TB	TB-LAMP	MGIT/ Xpert MTB/ RIF	101	69	0
2021	Wood (54)	Uganda	Prospective diagnostic evaluation	TB Hospital and Outpatient	Copan FLOQSwab	Front 2/3 tongue 15-20seconds	Presumptive TB	qPCR/ Culture	Sputum culture /Xpert Ultra	103	NR	NR
2022	LaCourse (55)	Kenya	Cross-sectional	Outpatient Clinic	OmniSwab	Left and Right buccal surface	Presumptive TB	qPCR	Sputum culture /Xpert MTB/ RIF	100	52	54
2022	Andama (56)	Uganda	Cross-sectional	Outpatient Clinic	COPAN FLOQswab	2 tongue swabs	Adults cough for greater than 2 weeks	Xpert Ultra	Xpert Ultra sputum	183	107	58
2022	Chang (57)	Uganda	Cohort	Outpatient Clinic	COPAN FLOQswab	15 sec dorsum of tongue	Symptoms of respiratory illness	Metagenomic	n/a	42	NR	NR

								sequencing assay				
2022	Kang (58)	Republic of Korea	Prospective diagnostic evaluation	Hospital	OMNIgene.ORAL OMR-110 kit	Palate, gum line and tongue dorsum	Clinically suspected of active PTB	SLIM assay	Culture positive (sputum, BAL)	272	174	1
2022	Shapiro (59)	South Africa	Prospective diagnostic evaluation	Hospital/Clinic	COPAN FLOQswab	Dorsum of tongue 15-20 seconds	Adult over 16 with TB symptoms	PCR	Sputum Xpert Ultra/ Culture	131	72	120
2022	Cox (60)	South Africa	Prospective diagnostic evaluation	Hospital	Purflock or COPAN FLOQswab	Swab tongue or inside of both cheeks	Children <15 presumptive PTB	Xpert Ultra	Induced sputum Xpert and culture	291	158	57
**Mouth wash**												
1955	Rogers (61)	USA	Diagnostic evaluation	Hospital	Mouthwash	Pharynx	Proven or presumptive TB	Culture	Sputum culture	43	NR	NR
1994	Evans (62)	UK	Case control	Outpatient clinic	Mouthwash	Pharynx	Active TB and controls	Lysed rRNA PCR	Sputum culture	39	NR	NR
2009	Davis (63)	Uganda	Diagnostic evaluation	Hospital	Mouthwash	Pharynx	Outpatients and inpatients	PCR	Sputum culture	127	NR	58
**Nasal swab**												
1996	Warndorff (64)	Malawi	Case control	Hospital	Cotton wool nasal swab	Nose	Inpatients and contacts	PCR	Sputum culture	52	NR	NR
**Saliva**												
2013	Shenai (65)	South Africa/Korea	Diagnostic evaluation	Hospital	Salivette saliva collector	Mouth	Adult PTB diagnosed patients	Xpert MTB/RIF	Sputum culture	26	NR	0

2015	Gonzalez (66)	Spain	Case control	Hospital	Spitting method into sterile container	Mouth	Adult presumptive TB	Culture	Sputum culture	32	21	NR
2022	Byanyima (67)	Uganda	Cohort	Hospital	Spitting method into sterile container	Mouth	Sputum Xpert Ultra positive for TB	Xpert Ultra	Sputum culture	82	50	18
2022	Hansen (68)	Germany	Case control	n/a	NR	Mouth	Patients with TB	qPCR	NR	17	NR	NR
**Other**												
2003	Eguchi (69)	Japan	Case control	Hospital	Unstimulated saliva in test tube	Mouth	Patients with TB	Culture/PCR	Culture	75	67	0
2012	Palakuru (70)	India	Case control	Outpatient clinic	Unstimulated saliva in sterile vial	Mouth	Pulmonary TB	PCR	Acid-fast bacilli positive	25	NR	0
2016	Balcells (71)	Chile	Cross sectional	Outpatient clinic	Bilateral nasopharyngeal COPAN FLOQswab	Nasopharyngeal	Adult contacts of smear positive cases	Xpert MTB/TIF	N/A	77	34	NR

NR: Not reported

S Table S6:

Full data table to show included reports and analysis of laryngeal swabs as sample type (all index and reference samples cultures full methodology in table S3).

Study	Comparison	TP	FN	FP	TN	Sens	Spec	Sens_Percentage	Spec_Percentage	LCI_sens	UCI_sens	LCI_spec	UCI_spec
1941_Nassau_Discharged 1940	Sputum culture	28	35	35	68	0.444444	0.660194	44.44444	66.01942	0.321741	0.567148	0.568722	0.751666

1941_Nassau_Inpatient 1940	Sputum culture	20	18	38	31	0.526316	0.449275	52.63158	44.92754	0.367559	0.685072	0.331906	0.566645
1948_Forbes_Inpatient	Gastric lavage	22	20	10	48	0.52381	0.827586	52.38095	82.75862	0.372764	0.674855	0.730371	0.924802
1948_Forbes_Outpatient	Gastric lavage	5	6	8	82	0.454545	0.911111	45.45455	91.11111	0.160288	0.748803	0.852316	0.969907
1948_Forbes_Mass radiography	Sputum culture	13	15	3	65	0.464286	0.955882	46.42857	95.58824	0.279556	0.649015	0.907072	1
1948_Hounslow_Hospital patients	Gastric lavage	18	14	21	140	0.5625	0.869565	56.25	86.95652	0.390618	0.734382	0.817543	0.921588
1950_Duggan_Inpatients and Outpatients both adults and children	Gastric lavage	27	3	5	65	0.9	0.928571	90	92.85714	0.792646	1	0.868239	0.988904
1950_Renoux_Sanatorium patients	Gastric lavage	19	4	4	29	0.826087	0.878788	82.6087	87.87879	0.67118	0.980994	0.767432	0.990144
1951_Armstrong_Hospital patients	Gastric lavage	84	15	105	763	0.848485	0.879032	84.84848	87.90323	0.777855	0.919115	0.857339	0.900726
1953_Chaves_Clinic patients	Gastric lavage	115	72	20	1211	0.614973	0.983753	61.49733	98.3753	0.545229	0.684718	0.976691	0.990816
1954_Frostad_TB	Gastric lavage	91	104	88	1217	0.466667	0.932567	46.66667	93.2567	0.396644	0.53669	0.918961	0.946173

hospital patients													
1955_Lind_Patients with lesions	Gastric lavage	11	13	2	95	0.458333	0.979381	45.83333	97.93814	0.258987	0.657679	0.951102	1
1955_Lind_Patients with lesions	Sputum culture	26	56	8	293	0.317073	0.973422	31.70732	97.34219	0.216353	0.417793	0.955251	0.991593
1955_Wallace_Inpatients	Sputum culture	37	14	13	73	0.72549	0.848837	72.54902	84.88372	0.60301	0.84797	0.773129	0.924545
1956_Engbaek_Outpatients	Gastric lavage	38	76	10	1107	0.333333	0.991047	33.33333	99.10474	0.246797	0.419869	0.985523	0.996571
1955_Tonge_Outpatients	Gastric lavage	52	78	2	333	0.4	0.99403	40	99.40299	0.315785	0.484215	0.98578	1
1962_Hsing_Hospital patients	Gastric lavage	264	90	46	907	0.745763	0.951731	74.57627	95.17314	0.700403	0.791123	0.938123	0.96534
1966_Pechacek_TB hospital patients	Homogenised 24 hours sputum	24	39	0	235	0.380952	1	38.09524	100	0.261035	0.50087	1	1
1966_Pechacek_TB hospital patients	Homogenised 24 hours sputum	28	29	0	170	0.491228	1	49.12281	100	0.361444	0.621012	1	1
1968_Lloyd, A. V. C._Inpatients	Gastric lavage	12	5	26	17	0.705882	0.395349	70.58824	39.53488	0.489282	0.922482	0.24921	0.541487
1950_Lundar_Inpatients	Gastric lavage	38	34	16	216	0.527778	0.931034	52.77778	93.10345	0.412462	0.643094	0.898427	0.963641

1952_Roald_ Inpatients	Gastric lavage	96	37	52	298	0.72 180 5	0.851 429	72.1804 5	85.1428 6	0.64564 7	0.79796 2	0.81416 7	0.88869
1952_Smeds rud_Inpatient s	Sputum culture	34	21	2	14	0.61 818 2	0.875	61.8181 8	87.5	0.48978 3	0.74658 1	0.71294 8	1

S7: Laryngeal swab reference standard meta-regression

A meta-regression was performed to model the difference in reference test (sputum culture or gastric lavage culture) as covariates. Firstly a separate meta-analysis was run for each reference test (Table S6), then a likelihood ratio test compared a further model (A) run without covariates to model (B) with equal variance and a covariate (A: without covariate sensitivity 59.2% [95% CI 51.2-66.7], specificity 91.8% [95% CI 86.0-95.4]), and there was statistical evidence (Chi2 = 10.277, 2 df, P = 0.006) of association of reference test type with sensitivity and/or specificity of laryngeal swabs. Two further models were then analysed one comparing sensitivity with reference sample as a covariate (C) and one of specificity (D). These showed there was a statistical significant difference in the modelled specificity but no difference in sensitivity (likelihood ratio comparing model B and C: Chi2 = 0.097, 2 df, P = 0.755, model B and D: Chi2 = 13.477, P = 0.0002 [Table S6]). Lastly a model allowing for unequal variances (E) was run which again showed a significant effect of the specificity of reference sample types (Fixed effect specificity gastric lavage Pr 1.68 x 10^-14, specificity sputum Pr 0.0015).

Table S7 to show model outputs when comparing gastric lavage and sputum culture when used as a reference test for laryngeal swabs.

Model	Gastric lavage		Sputum	
	Sensitivity (95% CI)	Specificity (95% CI)	Sensitivity (95% CI)	Specificity (95% CI)
Separate meta-analysis for each test	62.6 (52.9 to 71.4)	93.4 (87.8 to 96.5)	51.4 (40.1 to 62.6)	85.7 (66.5 to 94.8)
Test comparison assuming equal variances (model B)	62.4 (53.4 to 70.6)	93.4 (87.8 to 96.6)	51.6 (37.4 to 65.5)	85.5 (66.5 to 94.6)
Model comparing sensitivity (C)		94.3 (85.6 – 97.9)		78.9 (43.0 – 94.9)
Model comparing specificity (D)	61.9 (53.5-69.6)		47.0 (34.0 – 60.4)	
Test comparison allowing for unequal variances (model E)	62.6 (52.9 to 71.4)	93.4 (87.8 to 96.5)	51.4 (40.1 to 62.6)	85.7 (66.5 to 94.8)

Supplementary table S8:

Data table to show analysis of reports of NPA as sample type

Study / Index test analysis	Comparison (culture)	TP	FN	FP	TN	Sens	Spec	Sens_Percentage	Spec_Percentage	LCI_sens	UCI_sens	LCI_spec	UCI_spec
1998_Franchi/Culture	Gastric aspirate	17	7	2	38	0.708333	0.95	70.83333	95	0.526484	0.890183	0.882458	1
1998_Franchi/PCR	Gastric aspirate	13	4	5	42	0.764706	0.893617	76.47059	89.3617	0.563062	0.966349	0.805468	0.981766
2007_Owens/Culture	Induced sputum	16	3	5	64	0.842105	0.927536	84.21053	92.75362	0.678142	1	0.866364	0.988709
2012_Zar/Culture	Induced sputum	61	23	0	451	0.72619	1	72.61905	100	0.630831	0.82155	1	1
2013_Zar/Xpert MTB/RIF	Induced sputum	9	21	8	346	0.3	0.977401	30	97.74011	0.136015	0.463985	0.961919	0.992883
2019_Hanrahan/Xpert MTB/RIF	Induced sputum	0	2	2	90	0	0.978261	0	97.82609	0	0	0.948461	1
2019_Hanrahan/Culture	Induced sputum	0	0	1	93	NA	0.989362	NA	98.93617	NA	NA	0.968622	1
2019_Zar/Ultra	Induced sputum	21	19	5	150	0.525	0.967742	52.5	96.77419	0.370242	0.679758	0.939926	0.995558
2021_Song/Xpert MTB/RIF	Gastric aspirate MGIT	14	8	3	269	0.636364	0.988971	63.63636	98.89706	0.435347	0.83738	0.976559	1
2021_Song/MGIT	Gastric aspirate MGIT	18	4	4	268	0.818182	0.985294	81.81818	98.52941	0.65701	0.979353	0.970989	0.9996

S9: Nasopharyngeal reference standard meta-regression

A meta-regression was performed to model the difference in reference test (induced sputum culture or gastric aspirate culture) as covariates.

Firstly a separate meta-analysis was run for each reference test (Table S8), then a likelihood ratio test compared a further model (A) run without covariates to model (B) with equal variance and a covariate (A: without covariate sensitivity 73.2% [95% CI 57.7-84.5], specificity 92.0% [95%

CI 84.1-96.1]), and there was statistical evidence (Chi2 = 10.354, 2 df, P = 0.006) of association of reference test type with sensitivity and/or specificity of nasopharyngeal aspirates. Two further models were then analysed one comparing sensitivity with reference sample as a covariate (C) and one of specificity (D). These showed there was a statistical significant difference in the modelled specificity but no difference in sensitivity (likelihood ratio comparing model B and C: Chi2 = 0.82, 2 df, P = 0.364, model B and D: Chi2 = 9.42, P = 0.002 [Table S8]). Lastly a model allowing for unequal variances (E) was run which again showed a significant effect of the specificity of reference sample types (Fixed effect specificity gastric lavage Pr 6.18 x 10^-10, specificity sputum Pr 3.29 x 10^-15).

Table S9: to show model outputs when comparing gastric aspirate and induced sputum culture when used as a reference test for nasopharyngeal aspirates.

Model	Gastric lavage		Sputum	
	Sensitivity (95% CI)	Specificity (95% CI)	Sensitivity (95% CI)	Specificity (95% CI)
Separate meta-analysis for each test	73.2 (57.7 – 84.5)	92.0 (84.1-96.1)	56.3 (34.1-76.2)	98.4 (95.6-99.4)
Test comparison assuming equal variances (model B)	73.9 (45.8-90.5)	93.4 (33.3 – 99.8)	56.8 (38.8 – 73.1)	98.8 (91.8 – 99.8)
Model comparing sensitivity (C)		89.1(19.8 – 99.6)		99.0 (93.1-99.9)

Model comparing specificity (D)	77.4 (46.2 – 93.2)		55.8 (35.7 – 74.1)	
Test comparison allowing for unequal variances (model E)	73.2 (57.7. – 84.5)	92.0 (84.1 – 96.1)	56.3 (34.1 – 76.2)	98.4 (95.7 – 99.4)

Supplementary table S10:

Data table to show analysis of reports of NPA as sample type using clinical diagnosis as a reference test:

Study / Index test analysis	Clinical_comparison	Probable.PTB	True.positive.PTB	Definite.PTB	True.positive.DTB	No.TB	Combined.True.positive	FN	FP	TN	Sens	Spec	Sens_Percentage	Spec_Percentage	SE_sens	LCI_sens	UCI_sens	SE_spec	LCI_spec	UCI_spec
2007_Owens/Culture	Probable PTB	94	21	0	0	0	21	73	0	0	0.223404	NA	22.34043	NA	0.042961	0.1392	0.307609	NA	NA	NA
2018_Cakir/Culture	Probable PTB	40	5	0	0	0	5	35	0	0	0.125	NA	12.5	NA	0.052291	0.022509	0.227491	NA	NA	NA

2012_Zar/Culture	Probable and definite = positive, not = negative.	255	0	87	61	193	61	281	0	193	0.178363	1	17.83626	100	0.0207	0.13779	0.218935	0	1	1
2012_Zar/Xpert MTB/RIF	Probable and definite = positive, not = negative.	255	7	87	49	193	56	286	1	192	0.163743	0.994819	16.37427	99.48187	0.020001	0.124524	0.202961	0.005168	0.98469	1
2013_Zar/Xpert MTB/RIF	Probable and definite = positive, not = negative.	167	5	30	12	187	17	180	0	187	0.086294	1	8.629442	100	0.020006	0.047083	0.125506	0	1	1
2019_Hanrahan/Xpert MTB/RIF	Confirmed, unconfirmed, not	100	0	4	2	15	2	102	0	15	0.019231	1	1.923077	100	0.013467	-0.00716	0.045626	0	1	1
2019_Hanrahan/Culture	Confirmed, unconfirmed, not	100	0	4	1	15	1	103	0	15	0.009615	1	0.961538	100	0.009569	-0.00914	0.028371	0	1	1
2019_Zar/Ultra	Confirmed, unconfirmed, not	104	5	40	21	51	26	118	0	51	0.180556	1	18.05556	100	0.032054	0.117729	0.243382	0	1	1
2021_Song/Xpert MTB/RIF	Clinically suspected	294	17	0	0	0	17	277	0	0	0.057823	NA	5.782313	NA	0.013613	0.031142	0.084504	NA	NA	NA
2021_Song/MGIT	Clinically suspected	294	22	0	0	0	22	272	0	0	0.07483	NA	7.482993	NA	0.015345	0.044753	0.104907	NA	NA	NA

2020_Osorio/Xpert MTB/RIF	Clinically suspected	17	0	0	0	28	0	17	0	28	0	1	0	100	0	0	0	0	1	1
2020_Osorio/MGIT	Clinically suspected	17	4	0	0	28	4	13	0	28	0.235294	1	23.52941	100	0.102879	0.033651	0.436938	0	1	1

Supplementary table S11:

Data table to show analysis of reports of oral swabs as sample type.

Study / Index test analysis	Participants	Comparison	TP	FN	FP	TN	Sens	Spec	Sens_Percentage	Spec_Percentage	SE_sens	LCI_sens	UCI_sens	SE_spec	LCI_spec	UCI_spec
2015_Wood/PCR	TB clinic patients	GeneXpert positive on sputum	18	2	0	0	0.9	NA	90	NA	0.067082	0.768519	1	NA	NA	NA
2019_Luabeya/PCR	TB clinic patients	GeneXpert MTB/RIF plus MGIT	49	10	6	65	0.830508	0.915493	83.05085	91.5493	0.048845	0.734772	0.926245	0.03301	0.850793	0.980192
2019_Mesman/Xpert TB/Rif	TB clinic patients	MGIT sputum culture	15	18	0	0	0.454545	NA	45.45455	NA	0.086678	0.284656	0.624435	NA	NA	NA
2019_Nicol/PCR	Suspected pulmonary TB	IS Xpert MTB/Rif or sputum culture positive	17	23	22	103	0.425	0.824	42.5	82.4	0.078162	0.271802	0.578198	0.034062	0.757239	0.890761

2020_Flores/PCR	Suspected pulmonary TB	Bacteriologically confirmed	5	19	5	259	0.208333	0.981061	20.83333	98.10606	0.082898	0.045853	0.370814	0.008389	0.964617	0.997504
2020_Mesman/PCR	Culture confirmed TB	Culture positive	63	60	0	0	0.512195	NA	51.21951	NA	0.04507	0.423858	0.600532	NA	NA	NA
2020_Molina-Moya/PCR	Clinical diagnosis presumptive TB	MTBC culture	29	51	38	148	0.3625	0.795699	36.25	79.56989	0.053746	0.257157	0.467843	0.029563	0.737755	0.853643
2021_Ealand/MTBCg DNA	Young children less than equal 5 clinically diagnosed with TB	Gastric Aspirate culture/GeneXpert	4	2	7	22	0.666667	0.758621	66.66667	75.86207	0.19245	0.289464	1	0.079463	0.602874	0.914368
2021_Ealand/Spogliotyping		Gastric Aspirate culture/GeneXpert	2	4	12	17	0.333333	0.586207	33.33333	58.62069	0.19245	-0.04387	0.710536	0.091457	0.406951	0.765463
2021_Ealand/Auramine smear		Gastric Aspirate culture/GeneXpert	3	3	20	9	0.5	0.310345	50	31.03448	0.204124	0.099917	0.900083	0.085909	0.141963	0.478727
2021_Ealand/Culture		Gastric Aspirate culture/GeneXpert	0	6	0	29	0	1	0	100	0	0	0	0	1	1
2021_Song/TB-LAMP	> 16 years symptoms suggestive pulmonary TB	MGIT	33	5	8	55	0.868421	0.873016	86.84211	87.30159	0.054836	0.760942	0.9759	0.041948	0.790797	0.955235
2021_Wood/qPCR	Suspected TB	Culture	43	4	12	44	0.914894	0.785714	91.48936	78.57143	0.040702	0.835117	0.99467	0.054832	0.678243	0.893185
2021_Wood/Culture	Suspected TB	Sputum culture	82	50	0	9	0.621212	1	62.12121	100	0.042221	0.538458	0.703966	0	1	1

2022_LaCourse/qPCR	Suspected TB	Culture positive	12	7	16	65	0.631579	0.802469	63.15789	80.24691	0.110665	0.414676	0.848482	0.044237	0.715764	0.889174
2022_Andama/Xpert Ultra	Adults cough for greater than 2 weeks	Xpert Ultra/Solid and MGIT culture	42	16	0	125	0.724138	1	72.41379	100	0.058687	0.609111	0.839165	0	1	1
2022_Kang/SLIM assay	Clinically suspected of active PTB	Culture positive (sputum, BAL)	64	35	40	133	0.646465	0.768786	64.64646	76.87861	0.048048	0.552291	0.740638	0.032054	0.70596	0.831613
2022_Shapiro/PCR	Adult over 16	Sputum Xpert Ultra/Culture	25	39	2	65	0.390625	0.970149	39.0625	97.01493	0.060986	0.271092	0.510158	0.02079	0.9294	1
2022_Cox/Xpert Ultra	<15 presumptive PTB	Induced sputum Xpert and culture	20	70	1	200	0.222222	0.995025	22.22222	99.50249	0.043823	0.13633	0.308115	0.004963	0.985298	1

S12: Oral swab reference standard meta-regression

A larger variability in reference standard was seen in oral swab studies. A meta-regression therefore was carried out focused on sample type over culture v. Xpert as the model would have been overfitted with a larger number of covariates. Sixteen were classified as using sputum and four as gastric lavage. Firstly a separate meta-analysis was run for each reference test (Table S11), then a likelihood ratio test compared a further model (A) run without covariates to model (B) with equal variance and a covariate (A: without covariate sensitivity 56.7% [95% CI 44.3-68.2], specificity 91.3% [95% CI 81.0-96.3]), and there was statistical evidence (Chi2 = 6.16, 2 df, P = 0.046) of association of reference test type with sensitivity and/or specificity of oral swabs. Two further models were then analysed one comparing sensitivity with reference sample as a covariate (C) and one of specificity (D). These did not show a statistical significant difference in the modelled specificity or sensitivity

(likelihood ratio comparing model B and C: Chi2 = 2.01, 2 df, P = 0.156, model B and D: Chi2 = 2.30, 2 DF P = 0.130 [Table S11]). Lastly a model allowing for unequal variances (E) was run which showed a significant effect on specificity of reference sample types for sputum (Fixed effect specificity sputum Pr 9.27 x 10^-10).

Table S12 to show model outputs when comparing gastric aspirate and induced sputum culture when used as a reference test for oral swabs.

Model	Gastric lavage		Sputum	
	Sensitivity (95% CI)	Specificity (95% CI)	Sensitivity (95% CI)	Specificity (95% CI)
Separate meta-analysis for each test	33.0 (10.1 – 68.3)	80.1 (28.5 – 97.6)	59.5 (46.5 – 71.2)	93.4 (85.8 – 97.1)
Test comparison assuming equal variances (model B)	33.6 (11.3 – 66.7)	59.5 (46.3 – 71.4)	59.5 (46.3 – 71.4)	93.4 (85.4 – 97.2)
Model comparing sensitivity (C)		68.6 (32.4 – 90.9)		94.0 (86.5 – 97.4)
Model comparing specificity (D)	26.5 (8.3 – 59.0)		61.4 (48.1 – 73.2)	
Test comparison allowing for unequal	33.0 (10.1 – 68.3)	80.1 (28.5 – 97.6)	59.5 (46.5 – 71.2)	93.4 (85.8 – 97.1)

variances (model E)				

Supplementary table S13:

Other sample types

Study	Participants	Method.of.testing	Comparison	TP	FN	FP	TN	Sens	Spec	Sens_Percentage	Spec_Percentage	SE_sens	LCI_sens	UCI_sens	SE_spec	LCI_spec	UCI_spec
Mouth wash																	
1955_Rogers	Proven or suspected TB	Digestion, brought to 100ml and 10ml filtered	24 hour sputum culture	17	6	5	15	0.7391 3	0.75	73.91304	75	0.091561	0.559672	0.918589	0.096825	0.560224	0.939776
1955_Rogers			Gastric aspirate	1	2	0	88	0.333333	1	33.33333	100	0.272166	-0.20011	0.866778	0	1	1
1994_Evans	Active TB and controls	Lysed RNA PCR	Broncoscopy/Sputum culture	5	1	3	10	0.833333	0.769231	83.33333	76.92308	0.152145	0.535129	1	0.116855	0.540196	0.998266
2009_Davis	Outpatients and inpatients	PCR sec1	Sputum culture	55	20	6	46	0.733333	0.884615	73.33333	88.46154	0.051063	0.63325	0.833416	0.044305	0.797778	0.971453
Saliva																	
2015_Gonzalez	Adult suspected TB	Culture	Sputum culture	28	4	0	0	0.875	NA	87.5	NA	0.058463	0.760412	0.989588	NA	NA	NA

2022_Byanyima	Xpert Ultra positive for TB on sputum	Xpert Ultra	Sputum culture	70	8	2	1	0.897436	0.333333	89.74359	33.33333	0.034352	0.830106	0.964766	0.272166	-0.20011	0.866778
Other																	
2003_Eguchi	Patients with TB	Ogawa egg medium	Sputum culture	13	62	0	0	0.173333	NA	17.33333	NA	0.043709	0.087663	0.259004	NA	NA	NA
2003_Eguchi	Patients with TB	PCR	Sputum culture	51	1	0	0	0.980769	NA	98.07692	NA	0.019045	0.943441	1	NA	NA	NA
2012_Palakuru	Pulmonary TB	Nested PCR	AFB positive sputum	23	2	0	0	0.92	NA	92	NA	0.054259	0.813653	1	NA	NA	NA

SF1:

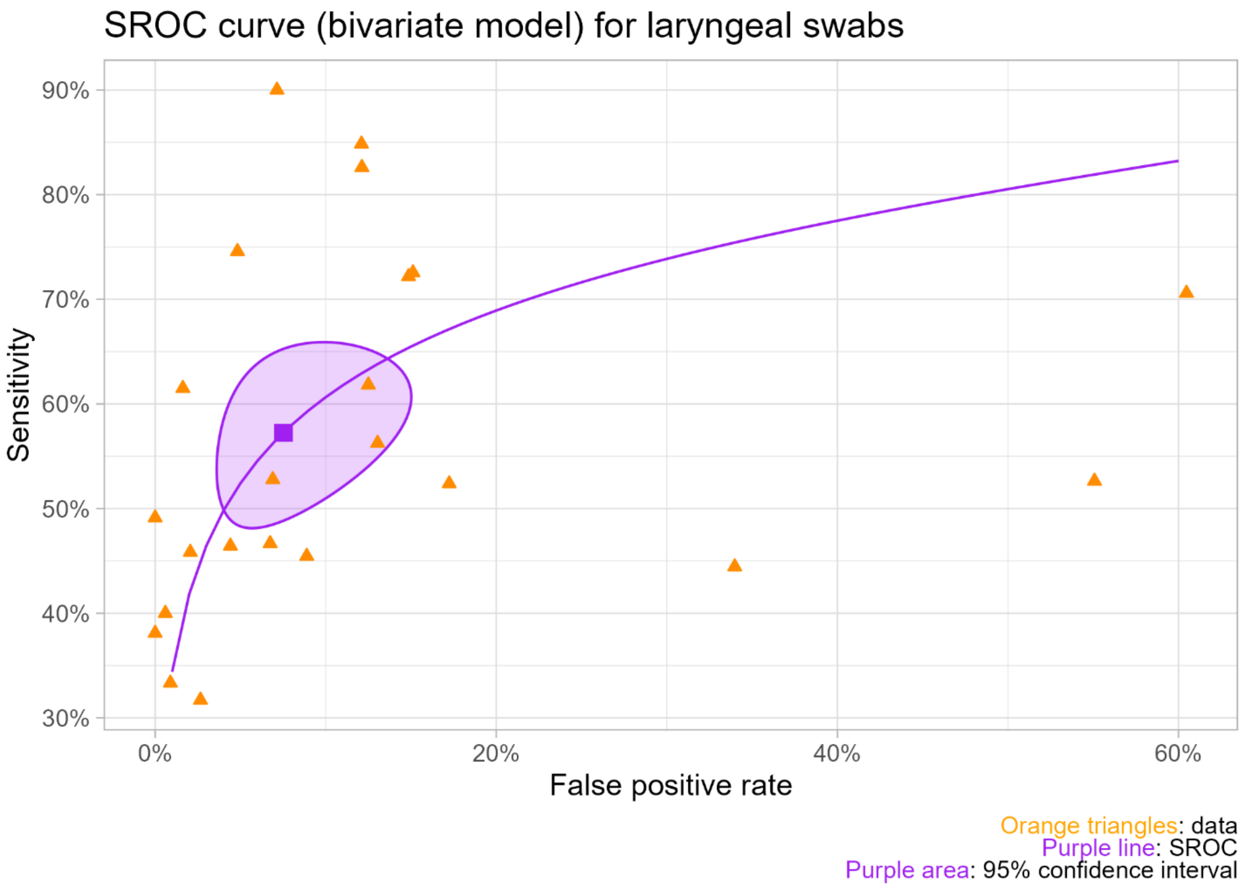
SROC curve (bivariate model) for laryngeal swabs
90%
80%
70%
60%
50%
40%
30%
Sensitivity
0%
20%
40%
60%
False positive rate
Orange triangles: data
Purple line: SROC
Purple area: 95% confidence interval

SF2:

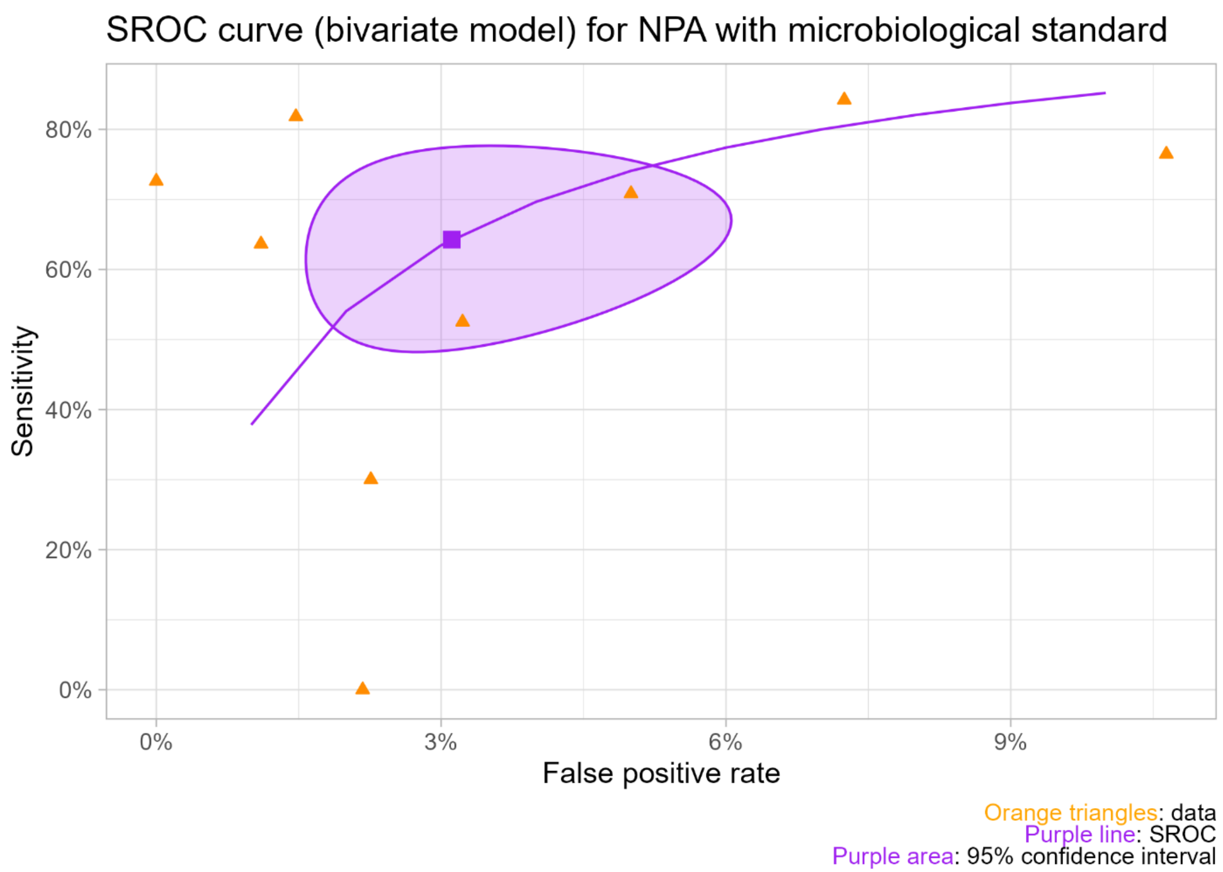
SROC curve (bivariate model) for NPA with microbiological standard
Sensitivity
80%
60%
40%
20%
0%
0%
3%
6%
9%
False positive rate
Orange triangles: data
Purple line: SROC
Purple area: 95% confidence interval

SF3:

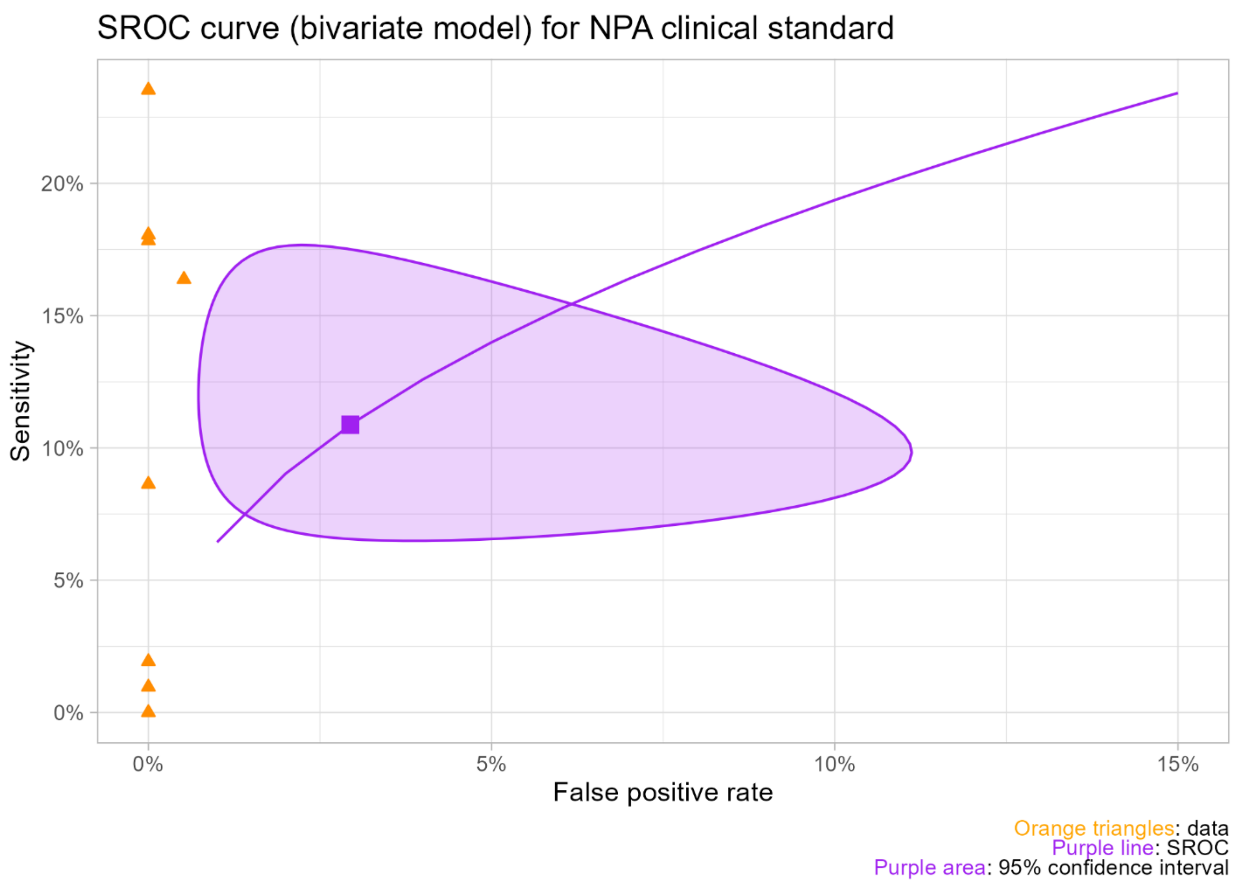
SROC curve (bivariate model) for NPA clinical standard
Sensitivity
20%
15%
10%
5%
0%
0%
5%
10%
15%
False positive rate
Orange triangles: data
Purple line: SROC
Purple area: 95% confidence interval

SF4:

SROC curve (bivariate model) for oral swabs

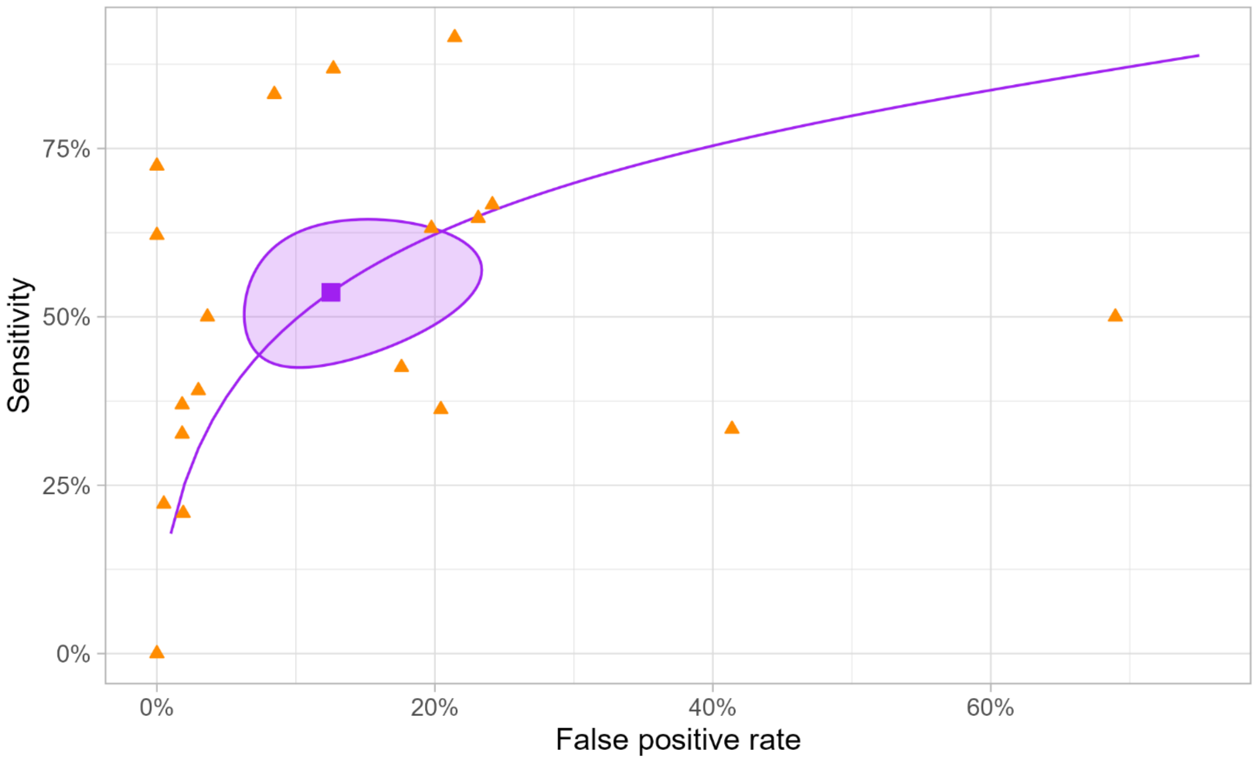

Orange triangles: data
Purple line: SROC
Purple area: 95% confidence interval

SF5: Bias of individual studies presented via each domain question and overall rating.

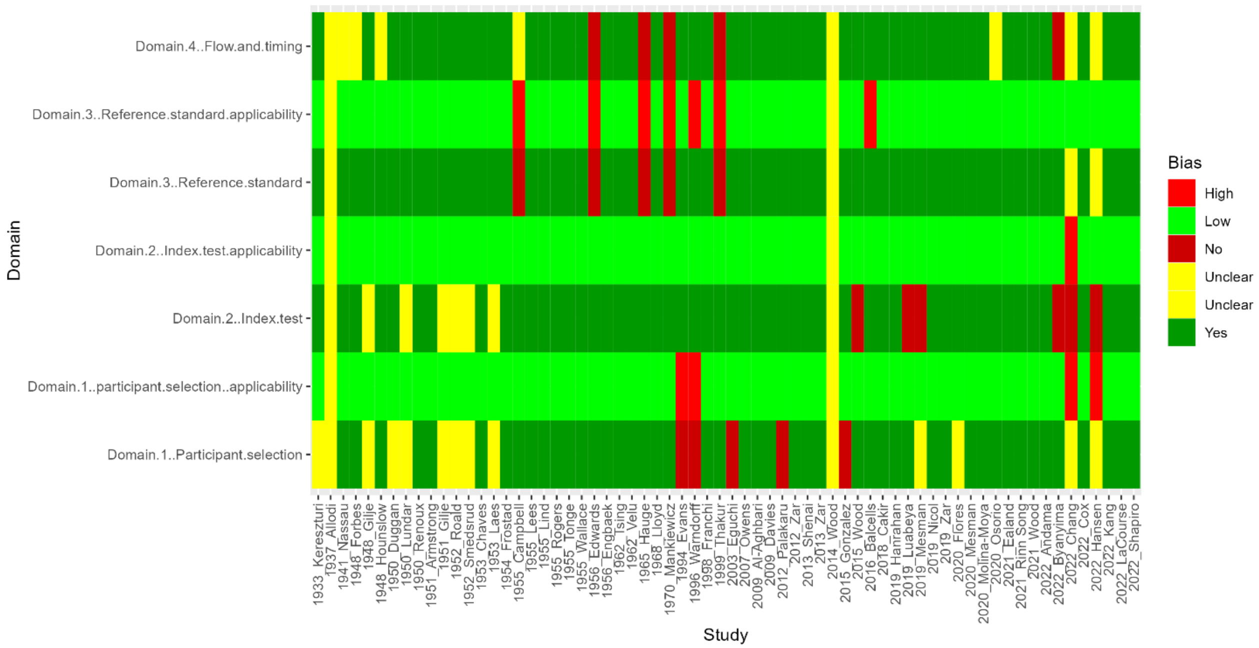

SF6: Bias of individual studies presented by domain.

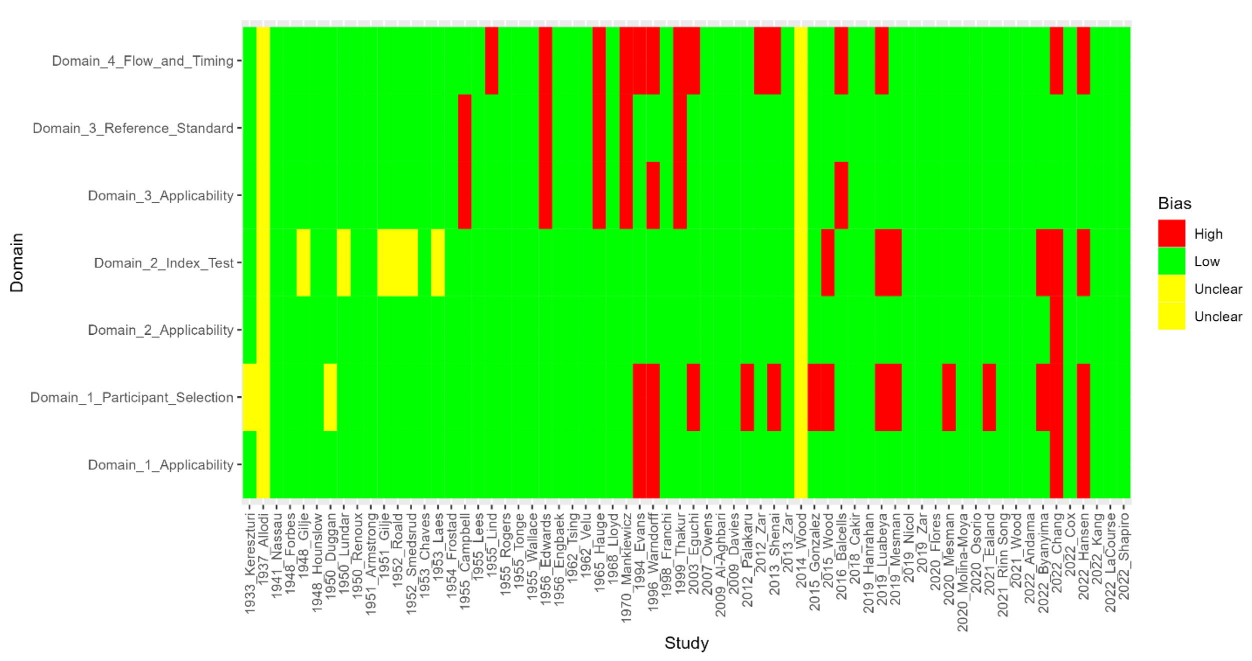

1. Kereszturi C. ACID-FAST BACILLI IN THE STOMACH LAVAGE AND FECES OF TUBERCULOUS CHILDREN. JAMA. 1933 May 13;100(19):1481.

2. Allodi A, Silorata AB, Marpillero P. 1937 Allodi et al PDF.pdf. Pubblicazone mensile dell'Ospedale Maria Vittoria di Torino. 1937 Dec;19(6):721.

3. Nassau E. The Culture of Tubercle Bacilli from Laryngeal Swabs. Proceedings of the Royal Society of Medicine. 1941;34(7):397–400.

4. Forbes VH GB: Hurford, JV: Smith, BJD: Springett. The Laryngeal Swab in Early and Convalescent Cases of Pulmonary Tuberculosis. Lancet. 1948;141–3.

5. Hounslow AG, Usher G. Examination for tubercle bacilli by gastric lavage and by laryngeal swab: a comparative study. Tubercle. 1948 Feb;29(2):25–31.

6. Duggan L M: Delamater. Laryngeal vs. gastric cultures in the detection of tubercle bacilli. Canadian Medical Association journal. 1950;62(1):54–6.

7. Renoux A GE: Francais. [Detection of the tubercle bacillus by culture of the laryngeal swab; comparison with the results of slide examinations and culture of gastric lavage fluid]. La Presse medicale. 1950;58(22):392–3.

8. Armstrong AR. The Laryngeal Swab Specimen in the Cultural Diagnosis of Pulmonary Tuberculosis. Canadian Medical Association Journal. 1951;65(6):575–8.

9. Szabo I. Experiences with the Cultivation of Tubercle Bacilli in Sula's Fluid Ascitic Medium. / Nase zkusenosti s kultivaci myco tbc v tekute ascitove pude podle Suly. Casopis Lekaru Ceskych. 1951;90(26):811–3.

10. Chaves D AD: Peizer, LR: Widelock. A Comparative Study of the Effectiveness of Laryngeal Swabs and Gastric Aspiration for the Detection of M. tuberculosis in Chest Clinic Patients. American Review of Tuberculosis and Pulmonary Diseases. 1953;67(5):598–603.

11. Frostad S. On the Reliability of the Laryngeal Swab Method and the Gastric Lavage Method for Tubercle Bacilli Demonstration. Acta Tuberculosea Scandinavica. 1954;29(3):214–22.

12. Campbell J AH: Kelly, JH: Banks. Laryngeal Swabs for the Detection of Tubercle Bacilli in Patients without Sputum. Medical Journal of Australia. 1955;2(21):852–4.

13. Lees AW, Miller TJR, Roberts GBS. BRONCHIAL LAVAGE FOR THE RECOVERY OF THE TUBERCLE BACILLUS. The Lancet. 1955 Oct;266(6894):800–1.

14. Lind A A: Lundin. The Laryngeal Swab Method for Detection of Tubercle Bacilli in Pulmonary Tuberculosis. A Comparison between Inoculation and Cultivation from Gastric Lavage, Sputum and Laryngeal Swab Specimens. Acta Tuberculosea Scandinavica. 1955;31(2):178–84.

15. Wallace AT, Ross JD, Schiller E. Laryngeal swabs for isolation of tubercle bacilli. British Journal of Tuberculosis and Diseases of the Chest. 1955 Jul;49(3):225–30.

16. Edwards JMB. The Storage of Laryngeal Swabs. Monthly Bull Ministry of Health & Pub Health Lab Service (directed by Med Res Council). 1956;15:95–8.

17. Engbaek V HC: Holm, S: Melton. Examination for Tubercle Bacilli by the Gastric Lavage and Laryngeal Swab Methods. A Gomparitive Study. Acta Tuberculosea Scandinavica. 1956;32(3):315–21.

18. Tonge PG JI: Hughes. A Comparative Study of Laryngeal Swabs and Gastric Lavage, in the Detection of Tubercle Bacilli. American Review of Tuberculosis and Pulmonary Diseases. 1956;73(6):930–9.

19. Hsing YT CT: Ma. A Comparative Study of the Efficacy of the Laryngeal Swab, Bronchial Lavage, Gastric Lavage, and Direct Sputum Examination Methods in detecting Tubercle Bacilli in a Series of 1,320 Patients. American Review of Respiratory Disease. 1962;86(1):16–20.

20. Velu TV S: Narayana, ASL: Subbaiah. A Comparison of the Results of Bacteriological Examination of a Sputum Collection and a Pair of Laryngeal Swab Specimens in Patients receiving Chemotherapy for Pulmonary Tuberculosis. Tubercle. 1962;43(1):1–10.

21. Kertay N, Marton S. [Comparative bacteriological investigations of the detection of Mycobacterium tuberculosis, using bronchial secretions obtained with the Marton method]. Acta Tuberc Pneumol Scand. 1962;41:161–8.

22. Hauge J HE: Schaanning. The Value of Repeated Laryngeal Swab Examinations during the Day. Acta Tuberculosea et Pneumol Scandinavica. 1965;46(2):141–3.

23. PechÁCek M. Comparative Study on Cultivation of Mycobacterium tuberculosis from Sputum, Bronchial Washout and Laryngeal Swabs. / Srovnávací studie o kultvaci Myco the ze sputa, bronchiálních výplachu a laryngeá lních výteru. Rozhledy v Tuberkulose. 1966;26(8):537–40.

24. Lloyd AVC. Bacteriological diagnosis of tuberculosis in children. A comparative study of gastric lavage and laryngeal swab methods. East African Medical Journal. 1968;45(3):140–3.

25. Mankiewi.E I : Shimro. LARYNGEAL SWAB METHOD FOR DETECTION OF TUBERCLE BACILLI IN PULMONARY TUBERCULOSIS. European Journal of Clinical and Biological Research. 1970;15(3):328-.

26. Thakur CA A: Coulter, JB: Zutshi, K: Pande, HK: Sharma, M: Banerjee, A: Richardson, K: Hart. Laryngeal swabs for diagnosing tuberculosis. Annals of tropical paediatrics. 1999;19(4):333–6.

27. Gilje A. Cultural Demonstration of Tubercle Bacilli by Laryngeal Swabbing. / Dyrking av larynxavstryk for påvising av tuberkelbasiller. Nordisk Medicin. 1948;40(44):1995–6.

28. Lundar J. [A comparison of laryngeal swabbing and gastric lavage as methods of demonstrating the tubercle bacillus]. Nordisk medicin. 1950;44(33):1306–7.

29. Gilje A. Demonstration of Tubercle Bacilli in Cultures : Comparison between Laryngeal Swabbing and Gastric Lavage. Nordisk Medecin. 1951;45(4):129–30.

30. Roald OK S: Thomassen. Reliability of the Laryngeal Swab Method for Tubercle Bacilli Cultures. / Larynxkulturers pålitelighet for påvising av tuberkelbasiller. Nordisk Medicin. 1952;48(47):1619–20.

31. Smedsrud K. Cultures from Laryngeal Swabs as a Method of assessing the Quantity of Bacilli in Pulmonary Tuberculosis. / Larynxkultur til kvantitativ vur-dering av basillutskillelsen ved tuberkulose. Deres betydning i det praktiske tuberkulosear-beid. Nordisk Medicin. 1952;47(19):623–6.

32. Laes T. Comparison of Tubercle Bacilli Cultivation from Throat Swabs and from Gastric Washings. Nordisk Medecin. 1953;49(8):289–90.

33. Franchi P LM: Cama, RI: Gilman, RH: Montenegro James, S: Sheen. Detection of Myobacterium tuberculosis in nasopharyngeal aspirate samples in children. Lancet (British edition). 1998;352(9141):1681–2.

34. Owens S, Abdel-Rahman IE, Balyejusa S, Musoke P, Cooke RPD, Parry CM, et al. Nasopharyngeal aspiration for diagnosis of pulmonary tuberculosis. Archives of Disease in Childhood. 2007 Apr 11;92(8):693–6.

35. Cakr M E: Özdemir, A: Daskaya, H: Umutoglu, T: Yüksel. The value of nasopharyngeal aspirate, gastric aspirate and bronchoalveolar lavage fluid in the diagnosis of childhood tuberculosis. Turkish Journal of Pediatrics. 2018;60(1):10–3.

36. Zar MP Heather J: Workman, Lesley: Isaacs, Washiefa: Munro, Jacinta: Black, Faye: Eley, Brian: Allen, Veronica: Boehme, Catharina C: Zemanay, Widaad: Nicol. Rapid molecular diagnosis of pulmonary tuberculosis in children using nasopharyngeal specimens. Clinical infectious diseases : an official publication of the Infectious Diseases Society of America. 2012;55(8):1088–95.

37. Zar MP Heather J: Workman, Lesley: Isaacs, Washiefa: Dheda, Keertan: Zemanay, Widaad: Nicol. Rapid diagnosis of pulmonary tuberculosis in African children in a primary care setting by use of Xpert MTB/RIF on respiratory specimens: a prospective study. The Lancet Global health. 2013;1(2):e97–104.

38. Hanrahan CF, Dansey H, Mutunga L, France H, Omar SV, Ismail N, et al. Diagnostic strategies for childhood tuberculosis in the context of primary care in a high burden setting: the value of alternative sampling methods. Paediatrics and International Child Health. 2019 Apr 3;39(2):88–94.

39. Zar MP HJ: Workman, LJ: Prins, M: Bateman, LJ: Mbhele, SP: Whitman, CB: Denkinger, CM: Nicol. Tuberculosis diagnosis in children using Xpert ultra on different respiratory specimens. American Journal of Respiratory and Critical Care Medicine. 2019;200(12):1531–8.

40. Song R, Click ES, McCarthy KD, Heilig CM, Mchembere W, Smith JP, et al. Sensitive and Feasible Specimen Collection and Testing Strategies for Diagnosing Tuberculosis in Young Children. JAMA Pediatr. 2021 May 3;175(5):e206069.

41. Osório DV, Munyangaju I, Muhiwa A, Nacarapa E, Nhangave AV, Ramos JM. Lipoarabinomannan Antigen Assay (TB-LAM) for Diagnosing Pulmonary Tuberculosis in Children with Severe Acute Malnutrition in Mozambique. Journal of Tropical Pediatrics. 2021 Jul 2;67(3):fmaa072.

42. Al-Aghbari N, Al-Sonboli N, Yassin MA, Coulter JBS, Atef Z, Al-Eryani A, et al. Multiple Sampling in One Day to Optimize Smear Microscopy in Children with Tuberculosis in Yemen. Pai M, editor. PLoS ONE. 2009 Apr 9;4(4):e5140.

43. Wood G R: Luabeya, A: Wilbur, A: Jones Engel, L: Filander, E: Hatherill, M: Cangelosi. Analysis of prevalent tuberculosis by using oral swab PCR. International Journal of Infectious Diseases. 2014;21:263–263.

44. Wood RC, Luabeya AK, Weigel KM, Wilbur AK, Jones-Engel L, Hatherill M, et al. Detection of Mycobacterium tuberculosis DNA on the oral mucosa of tuberculosis patients. Sci Rep. 2015 Aug;5(1):8668.

45. Luabeya AK, Wood RC, Shenje J, Filander E, Ontong C, Mabwe S, et al. Noninvasive Detection of Tuberculosis by Oral Swab Analysis. Miller MB, editor. J Clin Microbiol. 2019 Mar;57(3):e01847-18.

46. Mesman AW, Calderon R, Soto M, Coit J, Aliaga J, Mendoza M, et al. Mycobacterium tuberculosis detection from oral swabs with Xpert MTB/RIF ULTRA: a pilot study. BMC Res Notes. 2019 Dec;12(1):349.

47. Nicol MP, Wood RC, Workman L, Prins M, Whitman C, Ghebrekristos Y, et al. Microbiological diagnosis of pulmonary tuberculosis in children by oral swab polymerase chain reaction. Scientific Reports. 2019;9(1):1–5.

48. Flores JA, Calderón R, Mesman AW, Soto M, Coit J, Aliaga J, et al. Detection of Mycobacterium Tuberculosis DNA in Buccal Swab Samples from Children in Lima, Peru. Pediatric Infectious Disease Journal. 2020 Nov;39(11):e376–80.

49. Mesman MF AW: Calderon, RI: Pollock, NR: Soto, M: Mendoza, M: Coit, J: Zhang, ZB: Aliaga, J: Lecca, L: Holmberg, RC: Franke. Molecular detection of Mycobacterium tuberculosis from buccal swabs among adult in Peru. Scientific Reports [Internet]. 2020;10(1). Available from: <Go to ISI>://WOS:000603258300040

50. Molina-Moya B, Ciobanu N, Hernandez M, Prat-Aymerich C, Crudu V, Adams ER, et al. Molecular Detection of Mycobacterium tuberculosis in Oral Mucosa from Patients with Presumptive Tuberculosis. JCM. 2020 Dec 21;9(12):4124.

51. Lima F, Santos AS, Oliveira RD, Silva CCR, Gonçalves CCM, Andrews JR, et al. Oral swab testing by Xpert® MTB/RIF Ultra for mass tuberculosis screening in prisons. Journal of Clinical Tuberculosis and Other Mycobacterial Diseases. 2020 May;19:100148.

52. Ealand C, Peters J, Jacobs O, Sewcharran A, Ghoor A, Golub J, et al. Detection of Mycobacterium tuberculosis Complex Bacilli and Nucleic Acids From Tongue Swabs in Young, Hospitalized Children. Front Cell Infect Microbiol. 2021 Jun 14;11:696379.

53. Song Y, Ma Y, Liu R, Shang Y, Ma L, Huo F, et al. Diagnostic Yield of Oral Swab Testing by TB-LAMP for Diagnosis of Pulmonary Tuberculosis. IDR. 2021 Jan;Volume 14:89–95.

54. Wood RC, Andama A, Hermansky G, Burkot S, Asege L, Job M, et al. Characterization of oral swab samples for diagnosis of pulmonary tuberculosis. PLoS ONE. 2021;16(5 May 2021):1–13.

55. LaCourse SM, Seko E, Wood R, Bundi W, Ouma GS, Agaya J, et al. Diagnostic performance of oral swabs for non-sputum based TB diagnosis in a TB/HIV endemic setting. Hasnain SE, editor. PLoS ONE. 2022 Jan 13;17(1):e0262123.

56. Andama A, Whitman GR, Crowder R, Reza TF, Jaganath D, Mulondo J, et al. Accuracy of Tongue Swab Testing Using Xpert MTB-RIF Ultra for Tuberculosis Diagnosis. Journal of clinical microbiology. 2022 Jul 20;60(7):e0042122.

57. Chang A, Mzava O, Djomnang LAK, Lenz JS, Burnham P, Kaplinsky P, et al. Metagenomic DNA sequencing to quantify Mycobacterium tuberculosis DNA and diagnose tuberculosis. Scientific reports. 2022 Oct 10;12(1):16972.

58. Kang YA, Koo B, Kim OH, Park JH, Kim HC, Lee HJ, et al. Gene-Based Diagnosis of Tuberculosis from Oral Swabs with a New Generation Pathogen Enrichment Technique. Singh A, editor. Microbiol Spectr. 2022 Jun 29;10(3):e00207-22.

59. Shapiro AE, Olson AM, Kidoguchi L, Niu X, Ngcobo Z, Magcaba ZP, et al. Complementary Nonsputum Diagnostic Testing for Tuberculosis in People with HIV Using Oral Swab PCR and Urine Lipoarabinomannan Detection. Turenne CY, editor. J Clin Microbiol. 2022 Aug 17;60(8):e00431-22.

60. Cox H, Workman L, Bateman L, Franckling-Smith Z, Prins M, Luiz J, et al. Oral Swab Specimens Tested With Xpert MTB/RIF Ultra Assay for Diagnosis of Pulmonary Tuberculosis in Children: A Diagnostic Accuracy Study. Clinical Infectious Diseases. 2022 Dec 19;75(12):2145–52.

61. ROGERS DE, COOKE GM, MEYERS CE. The detection of tubercle bacilli in mouth wash specimens by the use of membrane filter cultures. American review of tuberculosis. 1955;71(3, Part 1):371–81.

62. Evans D, Goyal M, Taylor IK, Shaw RJ. Identification of M. tuberculosis ribosomal RNA in mouthwash samples from patients with tuberculosis. Respiratory Medicine. 1994 Oct;88(9):687–91.

63. Davis JL, Huang L, Kovacs JA, Masur H, Murray P, Havlir DV, et al. Polymerase Chain Reaction of *secA1* on Sputum or Oral Wash Samples for the Diagnosis of Pulmonary Tuberculosis. CLIN INFECT DIS. 2009 Mar 15;48(6):725–32.

64. Warndoff D, Glynn J, Fine P, Jamil S, de Wit M, Munthali M, et al. Polymerase chain reaction of nasal swabs from Tuberculosis patients and their contacts. International Journal of Leprosy. 1996;64(4):404–8.

65. Shenai S, Amisano D, Ronacher K, Kriel M, Banada PP, Song T, et al. Exploring Alternative Biomaterials for Diagnosis of Pulmonary Tuberculosis in HIV-Negative Patients by Use of the GeneXpert MTB/RIF Assay. J Clin Microbiol. 2013 Dec;51(12):4161–6.

66. González Mediero P G: Vázquez Gallardo, R: Pérez Del Molino, ML: Diz Dios. Evaluation of two commercial nucleic acid amplification kits for detecting Mycobacterium tuberculosis in saliva samples. Oral diseases. 2015;21(4):451–5.

67. Byanyima P, Kaswabuli S, Musisi E, Nabakiibi C, Zawedde J, Sanyu I, et al. Feasibility and Sensitivity of Saliva GeneXpert MTB/RIF Ultra for Tuberculosis Diagnosis in Adults in Uganda. Microbiology spectrum. 2022 Oct 26;10(5):e0086022.

68. Hansen J, Kolbe K, König IR, Scherließ R, Hellfritzsch M, Malm S, et al. Lipobiotin-capture magnetic bead assay for isolation, enrichment and detection of Mycobacterium tuberculosis from saliva. PloS one. 2022 Jul 15;17(7):e0265554.

69. Eguchi J, Ishihara K, Watanabe A, Fukumoto Y, Okuda K. PCR method is essential for detecting Mycobacterium tuberculosis in oral cavity samples. Oral Microbiology and Immunology. 2003;18(3):156–9.

70. Palakuru SK, Lakshman VK, Bhat KG. Microbiological analysis of oral samples for detection of Mycobacterium tuberculosis by nested polymerase chain reaction in tuberculosis patients with periodontitis. Dental Research Journal. 2012;9(6):6.

71. Balcells ME, Huilcaman M, Pena C, Castillo C, Carvajal C, Scioscia N, et al. M. tuberculosis DNA detection in nasopharyngeal mucosa can precede tuberculosis development in contacts. International Journal of Tuberculosis and Lung Disease. 2016;20(6):848–52.
